# Treatment Effects of Natural Products on Inflammatory Bowel Disease In Vivo and Their Mechanisms: Based on Animal Experiments

**DOI:** 10.3390/nu15041031

**Published:** 2023-02-18

**Authors:** Yaxi Zhou, Diandian Wang, Wenjie Yan

**Affiliations:** 1 College of Biochemical Engineering, Beijing Union University, Beijing 100023, China; 2 Beijing Key Laboratory of Bioactive Substances and Functional Food, Beijing Union University, Beijing 100023, China

**Keywords:** natural products, inflammatory bowel disease, therapeutic agents, mechanisms of action

## Abstract

Inflammatory bowel disease (IBD) is a chronic, non-specific inflammatory disease of the intestine that can be classified as ulcerative colitis (UC) and Crohn’s disease (CD). Currently, the incidence of IBD is still increasing in developing countries. However, current treatments for IBD have limitations and do not fully meet the needs of patients. There is a growing demand for new, safe, and highly effective alternative drugs for IBD patients. Natural products (NPs) are used in drug development and disease treatment because of their broad biological activity, low toxicity, and low side effects. Numerous studies have shown that some NPs have strong therapeutic effects on IBD. In this paper, we first reviewed the pathogenesis of IBD as well as current therapeutic approaches and drugs. Further, we summarized the therapeutic effects of 170 different sources of NPs on IBD and generalized their modes of action and therapeutic effects. Finally, we analyzed the potential mechanisms of NPs for the treatment of IBD. The aim of our review is to provide a systematic and credible summary, thus supporting the research on NPs for the treatment of IBD and providing a theoretical basis for the development and application of NPs in drugs and functional foods.

## 1. Introduction

Inflammatory bowel disease (IBD) is a chronic and specific inflammatory disease of the intestine, which can be divided into ulcerative colitis (UC) and Crohn’s disease (CD) depending on the disease manifestations. Among them, the lesions of UC are mostly located in the rectal and colonic areas, and the symptoms of onset are abdominal pain, blood in stool, weight loss, vomiting, etc. CD may occur in any part of the gastrointestinal tract, with clinical manifestations of abdominal pain, diarrhea, intestinal obstruction, accompanied by fever, nutritional disorders, and other manifestations. Furthermore, the course of CD is long, recurring, and rarely curable [1]. Today, many countries and regions are still plagued by IBD, with developed regions having a higher prevalence. IBD affects approximately 1.6 million people in the United States and up to 2 million people in Europe, and the prevalence of IBD is increasing in developing countries such as Asia, Africa, South America, and Eastern Europe [2,3].

Although the pathogenesis of IBD is complex and uncertain, numerous studies have confirmed that the development of IBD is related to genetics, environment, diet, intestinal barrier and immune response [1]. Genetic factors are important factors in the development of IBD, and people with a family history of the disease are more likely to develop IBD [4]. Epidemiological studies have also found that environmental factors play a key role in the pathogenesis of both UC and CD, and most of the environmental factors associated with IBD in life can mediate the pathogenesis of IBD by affecting the gut microbiota [5]. Persistent and severe imbalances of the gut microbiota can result in chronic inflammation of the gut as well as disruption of the integrity of the gut mucosa and intestinal barrier [6,7]. Immediately afterwards, the immune system is activated and the effector and regulatory cells in the intestinal mucosa become dysregulated, leading to the clinical manifestations of IBD [8].

Currently, there are a variety of therapeutic agents and treatments for IBD. Some of the good therapeutic drugs and treatments include amino salicylic acid agents, corticosteroids, immunomodulators, biological agents, stem cell transplantation, fecal microbiota transplantation, helminth therapy, and surgery [9,10]. However, current treatments do not meet the needs of all patients and have more pronounced side effects, while the failure of some drugs may exacerbate inflammation and intestinal damage in patients with IBD [11]. For example, amino salicylic acid preparations are often used to treat early and intermediate stage IBD, but their long-term side effects are high and poor patient compliance can lead to a high rate of IBD recurrence [12]. Similarly, long-term use of corticosteroids may be associated with an increased risk of death, and older patients are more drug dependent on corticosteroids [13]. IBD patients are in desperate need of new and effective therapeutic drugs. As a result, there is an urgent need to develop lower-cost, safer, and more effective anti-inflammatory drugs for IBD patients in order to overcome the limitations of current therapeutic drugs.

Because of their multiple biological activities, such as anti-inflammatory and antioxidant, natural products (NPs) may be a source for the development of new drugs with therapeutic effects in IBD. Numerous studies have found that NPs have in vivo therapeutic activity against experimental IBD models. Examples include natural flavonoids [14], natural terpenoids [15], glycosides [16], natural polyphenols [17], quinones [18], natural alkaloids [19], coumarins [20], and natural polysaccharides and bioactive peptides [21,22]. These NPs can improve and treat experimental IBD through multiple pathways.

In this paper, we first reviewed the pathogenesis of IBD as well as the current therapeutic approaches and therapeutic drugs. Furthermore, we used databases such as Google Scholar, PubMed, and the Web of Sciences to search for and summarize the therapeutic effects of 170 natural products from various sources on IBD, and we summarized their modes of action and therapeutic effects. To be more convincing, we excluded all relevant in vitro studies aimed at investigating the in vivo therapeutic effects of natural products on IBD. Finally, we investigated the potential mechanisms of NPs for IBD treatment. The goal of this review is to provide a systematic and credible summary, thereby supporting research into NPs for the treatment of IBD and providing a theoretical foundation for the development and application of NPs in drugs and functional foods.

## 2. Pathogenesis of IBD and Current Therapeutic Agents

### 2.1. The Pathogenesis of IBD

**Figure 1:**
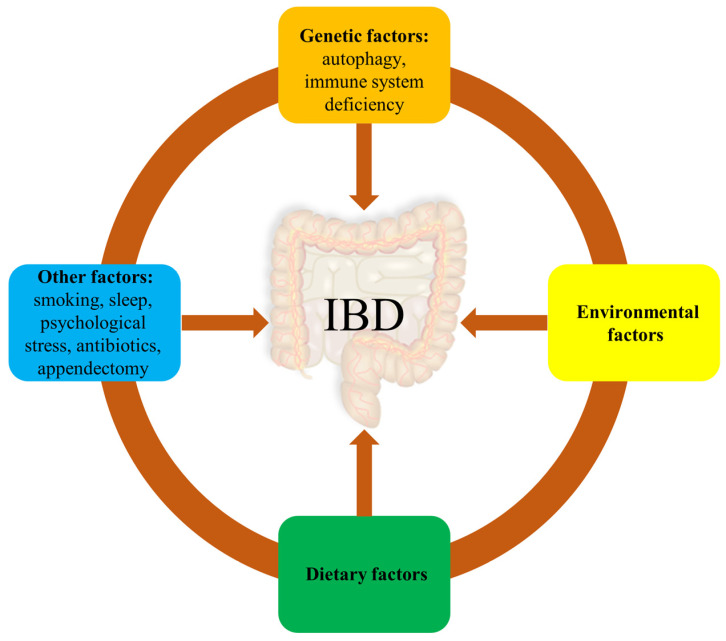
Causal factors of IBD.

To date, the exact cause of IBD is unknown, but numerous studies have found that the occurrence of IBD is associated with genetics, environment, gut microbes, hygiene, diet, sleep, mental health, smoking, antibiotic use, and post-surgical complications (Figure 1). Of these, genetics, environment, and diet are the three main factors [23].

#### 2.1.1. Genetic Factors

One of the primary causes of IBD is thought to be genetic factors. People with a family history of IBD are at a higher risk of developing the disease. IBD can develop at any age, according to research, but the prevalence is highest in early adulthood [24]. Using genome-wide association studies, 163 non-overlapping susceptibility gene loci were identified as early as 2012, including 30 CD-specific loci and 23 UC-specific loci [25]. Today, various studies have identified 242 susceptibility genes associated with IBD, of which NOD2 is the main susceptibility gene [4]. These susceptibility genes can significantly affect the autophagy, innate immunity, and adaptive immunity of the organism [26]. As a result, IBD is thought to be a disease with a strong genetic predisposition. Furthermore, it has been discovered that susceptibility alleles for IBD require other genetic and non-genetic factors to act in concert to manifest the disease state, making IBD pathogenesis more difficult and complex to comprehend [27,28]. In addition, it was found that for monozygotic twins, the concordance rate for UC was 10–15%, compared with 30–35% for CD. This suggested that although genetic factors are important for the development of IBD, non-genetic factors may play a more important role in UC than CD [24]. Genetic analysis of IBD suggested that the pathogenesis of IBD may involve variants of the innate and adaptive immune systems, as well as abnormalities of the intestinal epithelium [29]. In the future, it might be possible to treat IBD patients more effectively by choosing the right treatment modalities based on unique genetic factors by studying the genetic background of IBD and comprehending the genetic correlates of its development.

#### 2.1.2. Environmental Factors

In addition to genetic factors, the environment has a significant impact on the occurrence of IBD. First, geographic differences in IBD incidence have been linked. According to one study, women living in northern latitudes are more likely to develop UC with CD, which researchers attribute to people living in higher latitudes being less exposed to sunlight or UV radiation [30]. Moreover, since vitamin D has been shown to reduce inflammation and potentially affect the IBD process, vitamin D deficiency has also been suggested as a possible cause for the development of IBD [31]. People living at high altitudes may be vitamin D deficient due to insufficient sunlight exposure, making them more susceptible to IBD [32]. Besides that, studies have shown that people who are born and raised on livestock farms have a lower risk of developing IBD than those who live in cities. The study concluded that living on livestock farms as a child (before the age of five) is protective against the development of IBD in adulthood [33]. This may indicate that urban living is associated with developing CD and UC. In addition, environmental-related factors include passive smoking, environmental health, psychological stress, and exposure to drugs [34,35].

#### 2.1.3. Dietary Factors

Diet is considered a key factor in microbial dysbiosis and intestinal inflammation in IBD, and epidemiological studies have identified diet as a risk factor for IBD [36,37]. A recent article concluded that an incorrect diet can lead to immune system dysregulation, changes in intestinal permeability and mucosal layer, and microbial dysbiosis, which can lead to intestinal inflammation and increase the risk of IBD [38]. Currently, the six most commonly discussed dietary patterns in research are the Lactose-Free Diet, the Gluten-Free Diet, the Specific Carbohydrates Diet, the Anti-Inflammatory Diet, the Mediterranean Diet, and the low Fermentable Oligosaccharides, Disaccharides, Monosaccharides, and Polyols (FODMAPs) diet. All six dietary patterns have been found in studies to have potential effects on IBD activity [39]. According to the International Organization for the Study of Inflammatory Bowel Disease (IOIBD), patients with IBD should consume moderate to large amounts of vegetables and fruits and stop eating foods rich in additives. Designing a diet for IBD patients can achieve therapeutic benefits through diet, which not only avoids the long-term use of immunomodulators, but is also a low-cost and healthier form of treatment [36].

#### 2.1.4. Other Factors

In addition to these causative factors, the development of IBD has been shown to be associated with smoking, sleep deprivation, psychological stress, physical inactivity, overuse of antibiotics, and appendectomy. Studies have found that smoking is harmful to patients with CD, but some reports have illustrated the benefits of smoking for patients with UC [24]. However, it must be said that the nicotine in tobacco has been found to be used to improve experimental IBD [40]. Furthermore, sleep deprivation and psychological distress can also be associated with the development of IBD [41,42]. Regular exercise is thought to be beneficial for patients with IBD, while lack of exercise is associated with the pathogenesis of IBD [43]. Another factor influencing the development of IBD is antibiotic overdose. It was found that the risk of new-onset IBD and its subtypes may be increased when the cumulative amount of systemic antibiotic therapy is high, and that this risk is mainly associated with the dysbiosis of gut microbes due to overuse of antibiotics [44,45]. Additionally, appendectomy has been associated with the development of IBD. It has been shown that appendectomy increases the risk of developing UC and CD, regardless of whether the patient has appendicitis [46].

### 2.2. IBD Treatment Drugs and Therapeutic Methods

**Figure 2:**
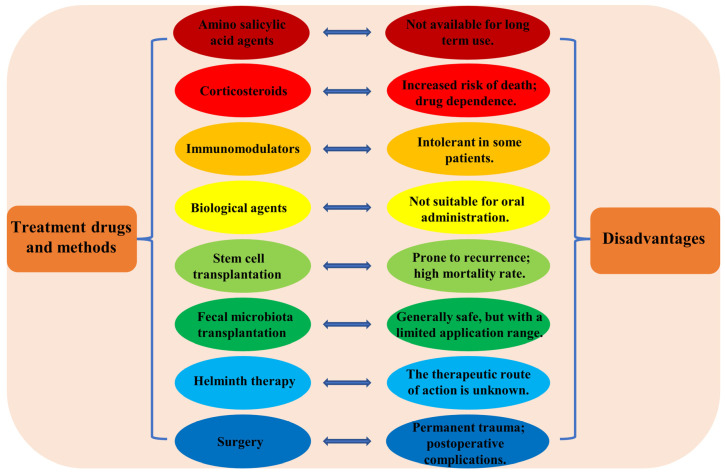
Therapeutic drugs and methods for IBD and their disadvantages.

Due to the complex pathogenesis of IBD, the therapeutic agents and treatments for IBD are complex and varied. Currently, some good therapeutic agents and treatments include amino salicylic acid agents, corticosteroids, immunomodulators, biological agents, stem cell transplantation, fecal microbiota transplantation, helminth therapy, and surgery. Among them, the mainstream methods are pharmacological treatments, such as amino salicylic acid agents, corticosteroids, immunomodulators, and biological agents. Although there is a wide range of therapeutic agents and methods for IBD, the therapeutic effect is not satisfactory and there are problems, such as poor patient compliance with medications, a high disease relapse rate, and therapeutic agents and treatments that are not universally applicable. Figure 2 demonstrates the IBD treatment drugs and methods and their disadvantages. Therefore, there is an urgent need to find low-cost, safer, and effective anti-inflammatory drugs for IBD patients to overcome the problems of existing therapeutic drugs.

#### 2.2.1. Amino Salicylic Acid Agents

5-Aminosalicylic acid (5-ASA) compounds, including salazosulfapyridine, mesalazine, and diazide-bonded 5-ASA, have been used as effective drugs for the treatment of IBD. According to studies, 5-ASA has a good therapeutic effect in patients with mild to moderate IBD, and the majority of these patients tolerate it well with few to no systemic side effects or gastrointestinal toxicity. However, for patients with severe illnesses, the therapeutic effect is not satisfactory [47]. Generally speaking, 5-ASA is safe to use for the treatment of IBD. However, there are some mild side effects such as headache, nausea, indigestion, flatulence, and diarrhea. However, some studies have shown that long-term use of 5-ASA has greater side effects and may lead to diseases such as pleurisy and myocarditis. Moreover, patient compliance can seriously affect its efficacy, and the risk of IBD recurrence is five times higher for people who do not adhere to 5-ASA treatment than for those who do [12].

#### 2.2.2. Corticosteroids

Corticosteroids are steroid hormones produced in the adrenal cortex, including glucocorticoids and salt corticosteroids. The use of corticosteroids to treat patients with IBD dates back to the 1950s. Although the effects of corticosteroids on IBD are obvious, corticosteroids should not be used for long periods of time and have significant side effects, such as an increased risk of death [48]. Studies have also confirmed that elderly patients with IBD may have a strong drug dependence on corticosteroids, which limits the possibility of their long-term use [13]. The representative drugs of corticosteroids are the first generation drugs prednisolone, methyl-prednisolone, hydrocortisone, second-generation drugs budesonide, budesonide MMX, and beclomethasone dipropionate [49]. At present, corticosteroids are only recommended for patients with 5-ASA refractory UC [50].

#### 2.2.3. Immunomodulators

The use of immunomodulators for the treatment of IBD has been shown to be a good therapeutic approach. The main immunomodulators currently used for the treatment of IBD are Thiopurines, Methotrexate, and Cyclosporine [51,52]. Thiopurines are derivatives of thiopurines and are mainly used to maintain long-term remission in patients with steroid-dependent IBD, especially UC [53]. It was found that approximately 10% of patients with IBD do not respond to thiopurines and that one-third of patients with IBD are intolerant to thiopurines. An alternative to thiopurine, methotrexate, may be used in these patients. Methotrexate is an antimetabolite that improves IBD by reducing T-cell activation, downregulating T-cell adhesion molecules, and blocking IL-1β binding to IL-1R on target cells [50]. However, methotrexate appears to be less effective than thiopurines and biological agents [54]. Another immunomodulatory agent is Cyclosporine. Cyclosporine improves IBD by inhibiting IL-2, TNF-α and IFN-γ production, T-cell proliferation, and the overall immune response of the body. but cyclosporine is only indicated for the remission of refractory acute severe UC [50,55].

#### 2.2.4. Biological Agents

Currently, the main biologics used in clinical IBD therapy are TNF-α inhibitors (Infliximab, Adalimumab, Certolizumab pegol, and Golimumab), anti-adhesion molecules (Natalizumab, Vedolizumab), anti-interleukin drugs (Ustekinumab, Risankizumab, and Brazikumab), Janus kinase inhibitors (Tofacitinib, Filgotinib, and Upadacitinib), and Sphingosine 1 Phosphate Receptor Modulator (Etrasimod and Ozanimod) [56]. For the treatment of IBD, biological agents provide a distinctive and different therapeutic approach that can significantly reduce intestinal inflammation in patients. However, biological agents are not recommended for oral administration because of their high sensitivity to the environment in the stomach and intestines and because they have safety concerns like toxicity [57]. Moreover, biologic agents are not universally applicable to all IBD patients, and the appropriate biologic agent needs to be provided to IBD patients on a case-by-case basis [58]. Therefore, following the use of corticosteroids, immunomodulators, and amino salicylic acid agents, biological agent therapy is still an option [56].

#### 2.2.5. Stem Cell Transplantation

Stem cell transplantation therapy appears to be used to improve clinical symptoms in patients with IBD when conventional therapies are ineffective, or even ineffective in the treatment of IBD. Stem cell transplantation therapy can be divided into hematopoietic stem cell transplantation (HSCT) and mesenchymal stem cell transplantation (MSCT) [59]. HSCT can be divided into autogenous and allogeneic transplantation. Autogenous transplantation is safer, but the possibility of recurrence is high because the graft comes from itself and does not change the patient’s genetic susceptibility at the genetic level. Allogeneic transplantation, on the other hand, can genetically alter the patient’s genetic susceptibility, but it has a high lethality rate. Despite the poor prospects of HSCT for the treatment of IBD, IBD patients with IL-10 gene defects seem to be cured by allogeneic transplantation [60]. The MSCT can repair damaged intestinal tissues by reducing the development of intestinal inflammation in IBD patients and by improving local microcirculation in the intestine [61]. Apart from a transient fever and a minimal risk of tumorigenesis, MSCT has not shown any other significant side effects. In addition, MSCT has low immunogenicity, does not require chemotherapy after transplantation, and has a low risk of serious complications [62]. Therefore, it is considered as a safer treatment [63]. Stem cell transplantation is a relatively new method of treating IBD, and more research is required to determine its safety and therapeutic effectiveness [59]. Therefore, stem cell transplantation is not yet a mainstream method used to treat IBD.

#### 2.2.6. Fecal Microbiota Transplantation (FMT)

Another non-mainstream approach is FMT. FMT is considered an alternative therapy for IBD and is only considered when the effects of drug therapy are not significant. FMT involves the input of a liquid fecal suspension obtained from a healthy donor into the gastrointestinal tract of patients with IBD in order to achieve microbiota transplantation and thus restore intestinal microbial function in patients with IBD [64]. The balance of intestinal flora in patients with IBD is usually disturbed and even bacterial infections may occur. For example, *C. difficile* infections are very common in IBD patients for the intestine, and FMT can treat *C. difficile* infections that are difficult to treat and have a high recurrence rate [65]. FMT is considered safe, especially in the treatment of IBD without *C. difficile* infection. To date, no serious adverse events have been reported with FMT [66]. FMT can be a safe and effective treatment for IBD when conventional therapies are not effective [67].

#### 2.2.7. Helminth Therapy

Initially, it was found that people infected with helminths had a lower probability and risk of developing IBD, and IBD was uncommon in areas where most people carried helminths. The beneficial effect of helminths on patients with IBD was later confirmed in animal experiments [68]. Later, *Trichuris suis* was also found to have a therapeutic effect on IBD in human experiments [69]. The interaction between host and parasite is complex, but numerous experimental and clinical studies have demonstrated that helminths can improve IBD by affecting intestinal luminal changes, modulating immune responses, regulating neuroendocrine responses, and producing immunosuppressive factors [70]. Notably, helminth therapy has a better ameliorative effect on T helper 1 (Th1)-dominated IBD. How the therapeutic effect of helminth therapy on IBD is exerted is unclear, but its therapeutic effect on IBD associated with Th1 makes it a viable means of IBD treatment. Currently, the main parasites that can parasitize humans and are potentially valuable for IBD are some nematodes and platyhelminths, such as roundworms, trematodes, flukes, and cestodes [70]. Helminth therapy is also not a mainstream therapy because the therapeutic action pathways are unclear, the action relationships are mixed, and most studies are limited to animal studies.

#### 2.2.8. Surgery

Surgery is the last treatment of choice for patients with IBD and is usually limited to very refractory patients. This is because surgery results in extensive resection of the intestine and permanent trauma to the organism. Surgery is generally not an option when drug therapy is feasible. Therefore, surgery is not a mainstream treatment for IBD [71]. However, in complex IBD, surgery is necessary when the damage to the body caused by continuous heavy drug therapy is greater than the effects of surgery. Surgery is a curative treatment for UC because it is concentrated in the colon and rectum. In contrast, unlike UC, the effect of surgery can usually only alleviate the complications and cannot cure it [72]. Of course, the need for surgery requires a comprehensive consideration of the patient’s actual situation to provide the best therapy tailored to IBD patients.

## 3. NPs with IBD Therapeutic Activity

**Figure 3:**
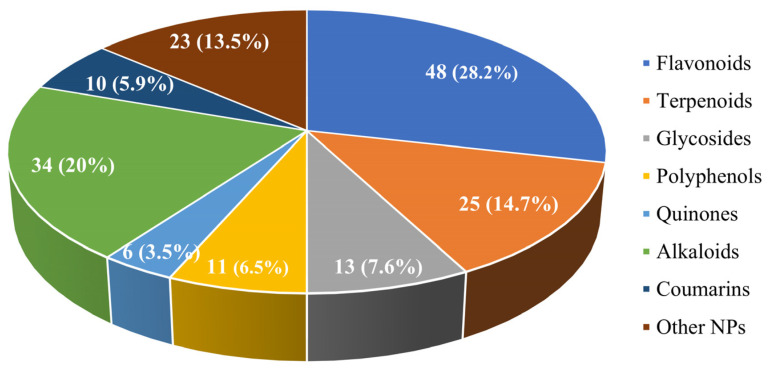
Number and percentage of different NPs with IBD therapeutic activity.

Based on the chemical structures and classes of compounds, we broadly classified natural products with IBD therapeutic activity into the following types: flavonoids, terpenoids, glycosides, polyphenols, quinones, alkaloids, coumarins, and polysaccharides and protein peptides. Here, we have grouped polysaccharides and protein peptides into other NPs. In Figure 3, we show the number and percentage of each type of NP. It can be seen that among all types of NPs, flavonoids and alkaloids have the largest percentages, at 28.2% and 20%, respectively. The therapeutic effects of each type of NP on experimental IBD models, as well as their sources, types, doses, and mechanisms of action, will be covered in detail in each of the following sections.

### 3.1. Flavonoids

**Figure 4:**
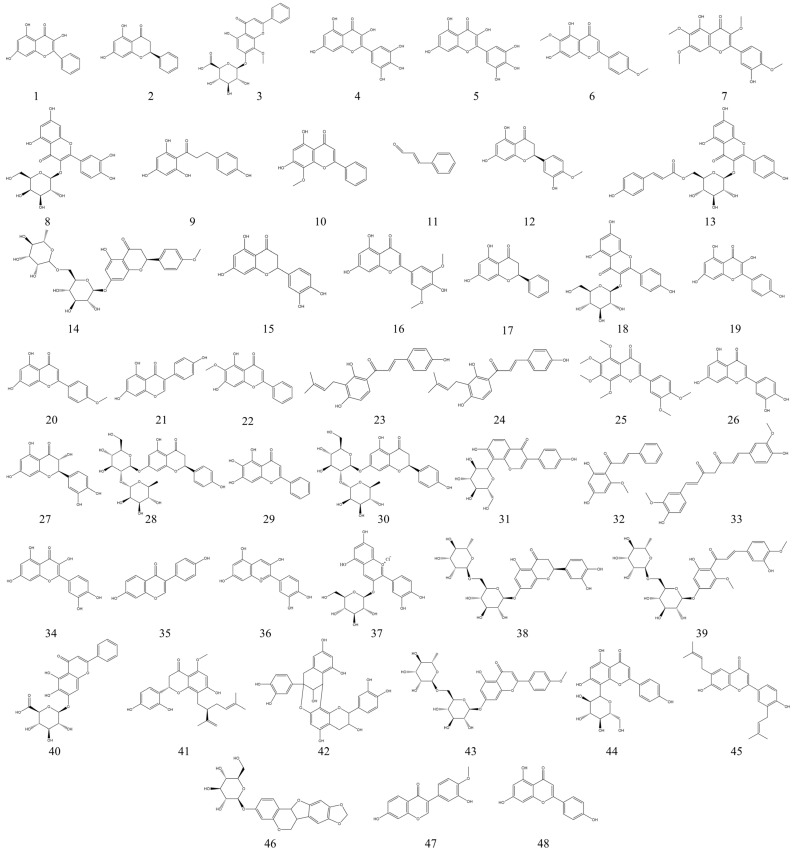
Structural formulae of flavonoids with IBD therapeutic effects.

Flavonoids are found in almost all green plants and are abundant in vegetables and fruits. Depending on their structures, flavonoids can be broadly classified as flavones, isoflavones, flavanols, flavanones, anthocyanins, etc. [73]. Flavonoids have a wide range of biological activities and are known to promote human health [74]. Numerous studies have shown that flavonoids from plants have therapeutic effects on inflammatory bowel diseases. For example, flavonoids from citrus can exert a modulatory effect on IBD by reducing the inflammatory response and inhibiting intestinal muscle contraction, significantly improving the pathological condition of experimental UC rats [75]. The fruit of *Lycium barbarum* is rich in flavonoids such as anthocyanins. Using anthocyanins from *Lycium barbarum* in DSS-induced UC mice, researchers found that anthocyanins from *Lycium barbarum* improved the symptoms of colitis in mice by affecting three aspects: inflammatory factors, increasing tight junction proteins, and regulating the intestinal microbiota [76]. Similarly, water-soluble isoflavones from soybean alleviated the symptoms of colitis in a mice colitis model by a potential mechanism of inhibiting inflammation by affecting the NF-κB pathway [77]. There is an extensive literature on the preventive and therapeutic effects of flavonoids on IBD. Flavonoids have been systematically described as therapeutic agents for IBD by investigators in recently published reviews [14,78]. Here, we summarized 48 flavonoids with therapeutic effects in experimental IBD models from the recently published literature. Of all the compounds we summarized, flavonoids accounted for 28.2% in terms of number. This higher percentage shows that flavonoids have a more pronounced therapeutic effect on IBD. The sources, types, doses, and mechanisms of action of these compounds are shown in detail in Table 1. Figure 4 shows the structural formulae of flavonoids with IBD therapeutic effects.

### 3.2. Terpenoids

**Figure 5:**
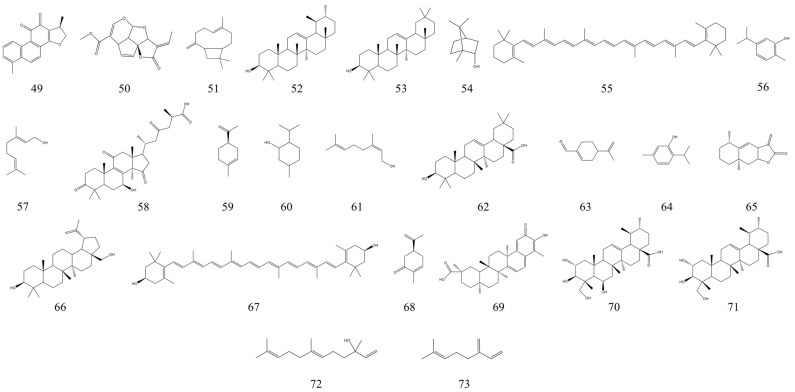
Structural formulae of terpenoids with IBD therapeutic effects.

Terpenoids are a group of naturally occurring source hydrocarbons widely found in plants and can be found in many plant bodies, especially conifers. Terpenoids can be classified as monoterpenes, sesquiterpenes, diterpenes, dibasic terpenes, triterpenes, tetraterpenes, and polyterpenes based on the number of isoprene structural units in the terpenoid structure. Many terpenoids have important physiological activities, such as being anti-inflammatory, anti-tumor, antibacterial, and antiviral, and are an important source for the study of natural products and the development of new drugs [132]. A recently published review summarized 281 terpenoids with anti-inflammatory activity and found that the powerful anti-inflammatory activity of terpenoids provides additional options for the development of anti-inflammatory drugs [15]. For IBD, some terpenes have also shown good therapeutic effects. Asiatic acid is a naturally occurring triterpenoid. Oral administration of Asiatic acid was found to significantly improve intestinal inflammation in mice with colitis and by inhibiting mitochondria-mediated activation of NLRP3 inflammatory vesicles [133]. Carvacrol, a phenolic monoterpene with anti-inflammatory and antioxidant activity, has been found to treat colitis in experimental mice. It was found to treat acetic acid-induced colitis in C57BL/6 mice by reducing the inflammatory response and oxidative damage [134]. Similarly, plumericin from *Himatanthus sucuuba* has been used by investigators as a candidate for the treatment of IBD due to its strong anti-inflammatory and antioxidant activities [135]. In Table 2, we summarized 25 natural terpenoids with therapeutic effects on IBD. It can be seen that the terpenoids with therapeutic effects on IBD mainly include monoterpenes, sesquiterpenes, triterpenes, and tetraterpenes. Figure 5 shows the structural formulae of terpenoids with IBD therapeutic effects.

### 3.3. Glycosides

**Figure 6:**
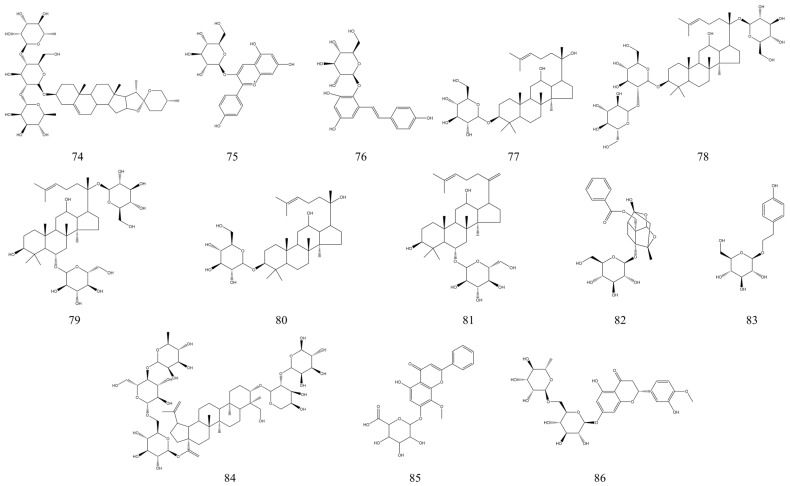
Structural formulae of glycosides with IBD therapeutic effects.

Glycosides are compounds formed by linking the end group carbon atoms of a sugar or sugar derivative to another class of non-sugar substances. Numerous studies have demonstrated that glycosides of natural origin have a wide range of biological activities, such as antiviral, anti-inflammatory, antitumor, and immunomodulatory [159,160]. In our search, we found that some natural glycosides have a better ameliorative effect on IBD; for example, Paeoniflorin [161], Salidroside [162], Wogonoside [163], Hesperidin [164], etc. It was found that glycosides from *Paeonia suffruticosa* significantly improved the clinical symptoms of IBD mice by reducing the inflammatory response [165]. In addition, total glucoside of Paeonia suffruticosa (TGP) was able to prevent IBD by modulating the IL-23/IL-17 axis and Th17/Treg homeostasis, and high doses of TGP had therapeutic effects on IBD similar to those of the therapeutic drug salicylazosulfapyridine [166]. In Table 3, we summarize 13 glycosides with in vivo therapeutic effects on experimental IBD. Figure 6 shows the structural formulae of the glycosides with therapeutic effects in IBD. It is worth mentioning that ginsenosides, the main active components of ginseng, have strong anti-inflammatory activity and are promising NPs for the treatment of IBD [167]. Ginsenoside Rd is a bioactive component of ginseng that stimulates the proliferation of endogenous stem cells. In a rat model of IBD, ginsenoside Rd stimulated the proliferation and differentiation of endogenous intestinal stem cells in rats, which, in turn, improved intestinal function [168]. In two IBD mouse models (DSS-induced and TNBS-induced), ginsenoside Rb1 could improve colitis by activating the endoplasmic reticulum-resident E3 ubiquitin ligase Hrd1 signaling pathway [16]. Not only that, ginsenoside Rg1 in ginseng can also regulate the intestinal microbiota of IBD mice to alleviate UC through microbial tryptophan metabolism [169]. Besides, ginsenoside Rh2 and ginsenoside Rk3 in ginseng have the same therapeutic and alleviating effects on experimental IBD [170,171]. Ginsenosides have significant therapeutic effects and low side effects and are likely to be used as a potential novel therapeutic agent for IBD [167].

### 3.4. Polyphenols

**Figure 7:**
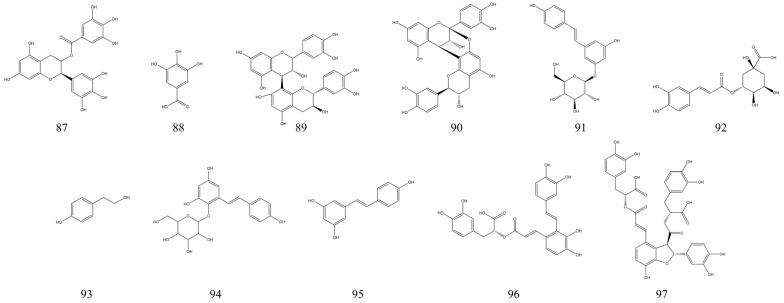
Structural formulae of polyphenolic compounds with IBD therapeutic effects.

Polyphenols are commonly found in vegetables and fruits, are secondary metabolites of many plants, and are also the most abundant source of natural antioxidants in the human diet. Numerous studies have shown that the intake of foods rich in polyphenols can be used to prevent and treat some common chronic diseases [177]. Polyphenols have a clear therapeutic effect on IBD due to their powerful antioxidant and anti-inflammatory properties [178]. For example, polyphenol extracts from spearmint (*Mentha spicata* L.) can attenuate inflammatory responses and colonic injury in IBD mice in vivo, and researchers have predicted that they may play an adjunctive role in the treatment of IBD patients [179]. Polyphenol extracts from Ripened Pu-erh tea (RPT, a famous traditional Chinese fermented tea) can ameliorate DSS-induced murine colitis. Researchers found that this may be due to the fact that RPT increased the level of short-chain fatty acids and PPAR-γ expression in the intestine [180]. Some other polyphenolic substances from plants have also been found to have IBD therapeutic activity, such as Black rice [181], Rock tea [182], etc. In Table 4, we summarize 11 polyphenolic compounds with in vivo therapeutic effects on experimental IBD. Figure 7 shows their structural formulae. Among these polyphenolic compounds, resveratrol showed a powerful therapeutic effect on IBD. Numerous studies have found that resveratrol can improve IBD pathology by modulating the intestinal microbiota of experimental IBD mice, reduce the inflammatory response, and alleviate intestinal mucosal barrier dysfunction in UC mice by enhancing autophagy of intestinal epithelial cells. It also inhibited the activation of PI3K/Akt pathway and reduced VEGFA gene expression to improve IBD [183,184,185]. In contrast, in-depth studies revealed that the therapeutic effect of resveratrol on IBD is mainly due to its alteration of the intestinal microbiota. In the presence of resveratrol, gut bacteria are also able to increase the production of short-chain fatty acids, and the gut microbiota has antioxidant and anti-inflammatory properties on the metabolites of resveratrol, which is very beneficial for the treatment of IBD [186]. It is certain that polyphenols of natural origin have potential therapeutic effects on IBD and can alleviate the symptoms of IBD through several pathways. Recently, a growing number of studies on the therapeutic effects of polyphenolic compounds on IBD have demonstrated that it is theoretically possible to develop drugs for the improvement of IBD using polyphenolic substances, and that polyphenols can be used as alternative or complementary therapies to conventional IBD treatments [17]. And some natural polyphenols with high activity may be included in the clinical trial phase, which may lead to the development of natural drugs with therapeutic effects in IBD [187].

### 3.5. Quinones

**Figure 8:**
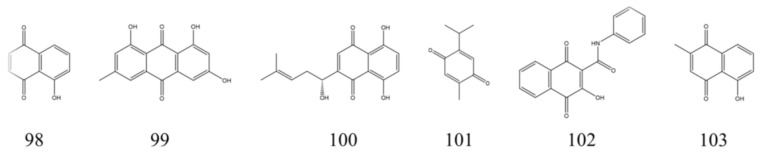
Structural formulae of quinones with IBD therapeutic effects.

Quinones are a class of plant secondary metabolites. Based on the number of benzene rings, quinones can be classified as anthraquinone, naphthoquinone, benzoquinone, and benzoquinone [199]. Several studies have found that quinones have IBD therapeutic activity. Here, we summarize six quinones that have in vivo therapeutic effects on experimental IBD (Table 5). Figure 8 shows their structural formulae. We found that among the four types of quinones, naphthoquinone exhibited more potent therapeutic effects on IBD. The investigators found that juglone, isolated from the green walnut husks of *Juglans mandshurica*, had in vivo therapeutic effects on DSS-induced UC mice. At a dose intervention of 1 mg/kg, juglone could treat UC in mice by modulating the intestinal microbiota and restoring Th17/Treg homeostasis [18]. Natural shikonin isolated from the root of *Lithospermum erythrorhizon* has also shown better effects when used to validate its therapeutic effects on UC due to its strong anti-inflammatory activity. After one week of treatment with shikonin at 25 mg/kg per day by gavage in experimental IBD model mice, their pathological symptoms were significantly reduced, probably by reducing inflammation and reversing intestinal mucosal damage [200]. Thymoquinone from *Nigella sativa*, which has antioxidant and anti-inflammatory properties, was found to reduce colonic inflammation in experimental IBD mice through its ability to modulate the Nrf2/Keap1 system [201]. Among the NPs that have been reported to have therapeutic effects in IBD, quinones are quantitatively modest. However, because they have therapeutic potential for IBD, they could also be a source of potential IBD therapeutic agents.

### 3.6. Alkaloids

**Figure 9:**
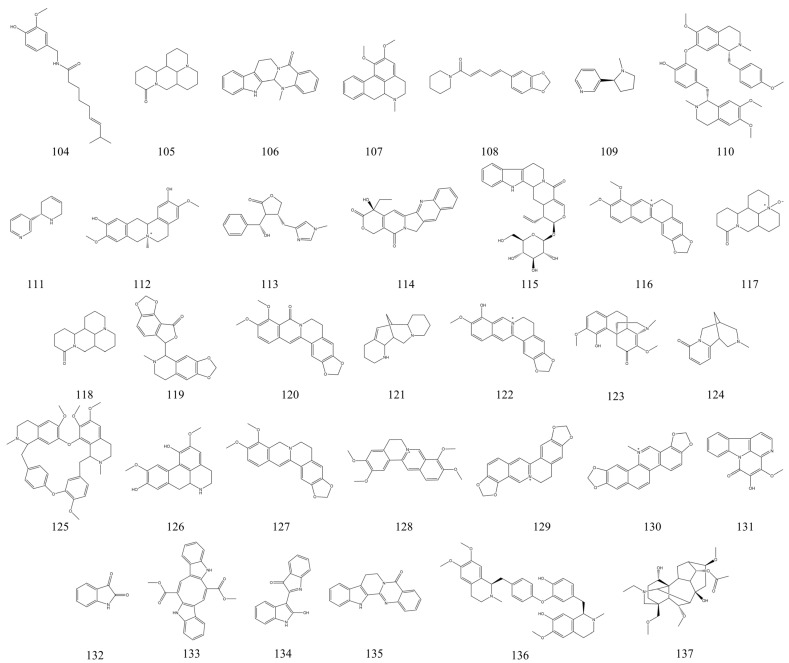
Structural formulae of alkaloids with IBD therapeutic effects.

Alkaloids are a type of nitrogenous organic compound derived from amino acids that can be found in plants, animals, and microorganisms [204]. Alkaloids are usually classified as indoles, isoquinolines, and pyridine alkaloids. Alkaloids of natural origin have been shown to have a variety of pharmacological activities, such as anti-inflammatory, immunomodulatory, and anti-cancer [19]. Recently, numerous studies have reported the therapeutic effects of different sources of natural alkaloids on experimental IBD [205]. In this paper, we summarize 34 natural alkaloids with IBD therapeutic effects (Table 6), and their chemical structural formulae are presented in Figure 9. Quantitatively, the number of alkaloids with IBD therapeutic activity is second only to the flavonoids. This suggests that among the many NPs, the therapeutic potential of alkaloids for IBD is enormous. Berberine, an isoquinoline alkaloid, was found to have a significant therapeutic effect on experimental IBD. Its potential mechanism of action is to improve clinical symptoms in mice with experimental IBD by modulating the intestinal microbiota and protecting the intestinal mucosal barrier [206]. Further studies revealed that berberine may have a therapeutic effect by modulating glial-intestinal epithelial cell-immune cell interactions, thereby improving intestinal neuroinflammation [207]. Piperine from *Piper longum* Linn. is a natural alkaloid of plant origin with a long history of medicinal use. In a mouse IBD model, piperine exhibited potent IBD therapeutic activity. At a dose of 10 mg/kg/day, piperine significantly reduced DAI scores and reduced the inflammatory response by inhibiting the IκB-α/NF-κB signaling pathway in mice [208,209]. Therefore, piperine may be used as an anti-inflammatory agent for the treatment of IBD. Camptothecin, a quinoline alkaloid extracted from *camptotheca acuminata*, was also found to have therapeutic effects in mice with experimental IBD. Camptothecin may improve the expression of cellular inflammatory factors in mice with IBD by modulating clinical symptoms in IBD mice by modulating the expression of cellular inflammatory factors [210]. A recent review illustrated the great therapeutic potential of alkaloids of natural origin for UC [19]. This article suggested that the potential mechanisms of natural alkaloids for treating UC are closely related to their regulation of oxidative stress, immune response, intestinal flora, and improvement of intestinal barrier function.

### 3.7. Coumarins

**Figure 10:**
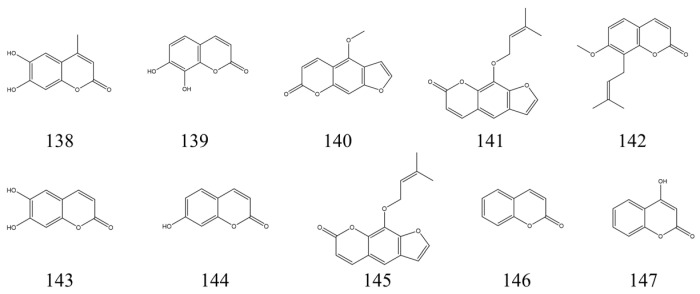
Structural formulae of coumarin analogues with IBD therapeutic effects.

Coumarins are widely present in different parts of the plant and are secondary plant metabolites consisting of a thickened benzene ring and an α-pyrone ring. The mechanism of action of coumarins in experimental IBD models is similar to that of other NPs, such as flavonoids and polyphenols. So far, most of the therapeutic effects of coumarins on IBD have been achieved mainly by reducing inflammatory responses and modulating oxidative stress and immune responses. A few studies have also reported that coumarins act through other signaling pathways, such as NF-κB and PPAR-γ Signaling Pathways, MAPK Signaling Pathway, HIF-1α Signaling Pathway, etc. [20]. In Table 7, we summarize 10 natural coumarins with IBD therapeutic effects, and their chemical structural formulae are presented in Figure 10. Osthole is a natural coumarin-like compound isolated from *Cnidium monnieri*. Previous studies have shown that osthole has anti-inflammatory activity and a protective effect on the intestinal tract of mice with experimental IBD [247,248]. Further studies revealed that osthole could treat experimental IBD by blocking the activation of NF-κB and MAPK/p38 pathways, as well as reducing the expression of inflammatory mediators [249]. Umbelliferone is a coumarin derivative with anti-inflammatory and antioxidant effects. Umbelliferone was found to have a significant ameliorative effect on acetic acid-induced UC. Umbelliferone not only significantly ameliorated histological damage in rats, but also treated UC by exerting anti-inflammatory and antioxidant effects. Its potential mechanism is to promote the SIRT1/PPARγ signaling pathway to protect UC rats [250].

### 3.8. Polysaccharides

Natural polysaccharides have powerful bioactivities, especially anti-inflammatory activity [259]. In recent years, natural polysaccharides have received much attention because of their high activity, safety, and easy access [21]. Notably, a large number of studies have reported the therapeutic and ameliorative effects of natural polysaccharides on IBD [260]. For example, a polysaccharide (EP-1) isolated and purified from the monkey head mushroom could alleviate acetic acid-induced UC. It was found that EP-1 ameliorates UC symptoms by modulating the intestinal microbiota and increasing the content of short-chain fatty acids in the intestine [261]. Water-soluble polysaccharides isolated from *Auricularia auricular-judae* can be used to treat experimental IBD by protecting the intestinal barrier and regulating the intestinal microbiota in mice [262]. An active polysaccharide from Astragalus has also been shown to have an ameliorative effect on experimental IBD. Using a DSS-induced acute UC mouse model to investigate its mechanism of action, it was shown that Astragalus polysaccharide could improve experimental IBD by inhibiting the NRF2/HO-1 pathway and regulating Tfh/Treg cell homeostasis [263,264]. Additionally, polysaccharides from *Dictyophora indusiata* may also improve the clinical symptoms of experimental IBD by modulating intestinal microbiota and inflammation-related signaling pathways [265]. Natural polysaccharides have a very good effect on the regulation of the intestinal microbiota, according to the extensive literature on the improvement of IBD by natural polysaccharides [266]. On the one hand, natural polysaccharides can restore the imbalance of the intestinal microbiota and protect the intestinal barrier. On the other hand, the metabolism of polysaccharides by the intestinal microbiota produces large amounts of short-chain fatty acids and other metabolites, especially tryptophan, which are also well known to improve experimental IBD [267]. Short-chain fatty acids have been found to play an important role in maintaining intestinal function and intestinal epithelial cell homeostasis [268]. Most of the natural polysaccharides with ameliorative effects on experimental IBD are of plant origin. Studies have shown that natural plant polysaccharides are not only low in toxicity and high in activity, but also have a very positive effect on the relief of IBD. Among them, natural plant polysaccharides rich in galactose and mannose are more effective in the treatment of IBD [269]. A recent study had discussed the ameliorative effects and mechanisms of action of natural polysaccharides on IBD. It was found that natural polysaccharides exerted their ameliorative effects on IBD mainly in terms of improving clinical symptoms of IBD, repairing colonic tissues, reducing oxidative stress, decreasing inflammatory response, immunomodulation, and regulating intestinal flora [21]. In Table 8, we summarized 18 recently published natural polysaccharides with in vivo therapeutic effects on experimental IBD models and their potential mechanisms of action.

### 3.9. Natural Proteins and Active Peptides

Protein peptides have been found to have a variety of active functions, and several peptides have been found to improve intestinal inflammation and have therapeutic effects on experimental inflammatory bowel disease [286,287,288]. Evidence for the use of bioactive peptides in reducing intestinal inflammation has been investigated through the use of in vivo models and clinical trials [22]. In Table 9, we summarized the therapeutic effects of natural proteins and active peptides on experimental IBD. Small peptides obtained from plants have alleviating effects on DSS-induced inflammatory bowel disease, such as the tripeptide VPY from soybean and the dipeptide pyroGlu-Leu from wheat [287,288]. Phycocyanin has been found to possess a variety of physiological activities. It was found that phycocyanin could improve colitis in mice by protecting the intestinal epithelial cell barrier and exerting anti-inflammatory and antioxidant effects [289]. Moreover, recent studies have found that certain protein peptides from animal sources also have the same function of improving colitis; for example, bee venom peptides [290] and yellowtail protein hydrolysates [291]. However, the unstable nature of peptides and their easy inactivation after digestion in the gastrointestinal tract make the treatment of inflammatory bowel disease with peptides more challenging [292]. Today, the development of protein modification and encapsulation techniques has allowed the protection of functional peptide activity. For example, when annexin A1-derived tripeptide MC-12 was grafted onto the disulfide-rich peptide linaclotide, its stability and biological activity were improved [292]. When MC-12 was transplanted into a sunflower trypsin inhibitor ring scaffold, in vivo experiments revealed that it significantly improved acute colitis in mice and exhibited greater stability [293]. Natural proteins have a wide range of biological activities. Oral administration, on the other hand, has a poor pharmacokinetic profile and low bioavailability. Proteins of natural origin may be a promising source of drugs for the treatment of inflammatory bowel disease if their activity is protected by appropriate methods [294].

## 4. Major Pathways of Action of NPs in the Treatment of IBD

**Figure 11:**
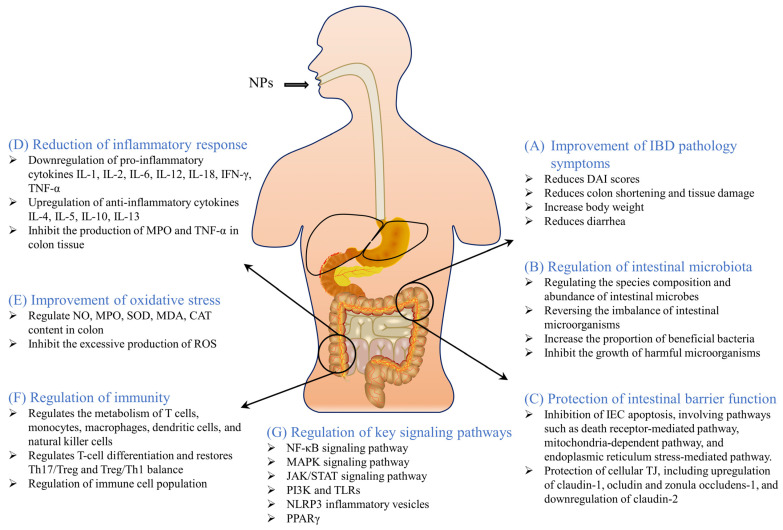
Mechanism of therapeutic action of NPs for IBD. (**A**–**F**) are the main pathways of action of NPs for the treatment of IBD; (**G**) is a relevant signaling pathway for NPs to improve IBD.

The therapeutic activity of NPs for IBD was shown categorically in the previous section. Here, we will elaborate on the main pathways of action of NPs for the treatment of IBD. The results of multiple in vivo experimental IBD models suggested that the ameliorative and therapeutic effects of NPs on experimental IBD are exerted mainly through the following six aspects. Figure 11 demonstrates the mechanism of action of NPs for IBD treatment.

### 4.1. Improvement of IBD Pathology Symptoms

The main clinical symptoms of IBD are bloody stools, abdominal pain, diarrhea, and weight loss [296]. The pathological conditions in the chemical agent-induced IBD model in mice or rats are generally characterized by colonic inflammation, weight loss, diarrhea, bloody stools, and colon shortening, which are very similar to the clinical symptoms of human IBD [297]. The pathological evaluation of IBD is based on the Disease Activity Index (DAI) score, which is the average of weight loss, fecal status, and blood stool scores, and is used to evaluate the severity of IBD [297]. All 170 NPs that we summarized as having ameliorative and therapeutic effects on experimental IBD improved the pathological symptoms of experimental IBD. For example, alpinetin, a natural flavonoid isolated from *Alpinia katsumadai Hayata*, significantly improved the symptoms of DSS-induced IBD in mice. This was demonstrated by a reduction in DAI scores and histopathology scores and reversal of colonic shortening [84]. Similarly, the natural polyphenol gallic acid significantly reduced DAI and colonic shortening in UC mice and reduced pathological damage to colonic tissue [189]. Caulerpin, a natural alkaloid, has been shown to have an ameliorative effect on experimental IBD. It was found that caulerpin given to IBD mice at a dose of 4 mg/kg per day significantly promoted the reduction of DAI and attenuated shortening and damage to the colon [241]. In addition, sanguinarine had the same effect of improving pathological symptoms in IBD mice. Compared to acetic acid-induced controls, sanguinarine treatment significantly reduced mortality and improved weight loss, DAI scores, colonic weights, and histological scores in mice [238]. Astragalus polysaccharide was also able to significantly improve clinical signs in IBD mice by increasing survival, DAI scores, body weight, colon length, rate of weight change, and histopathological damage to the colon [264]. *Morinda officinalis* root polysaccharide treatment not only significantly reduced diarrhea, weight loss, colonic shortening, and histological damage in UC mice, but also inhibited spleen swelling and structural changes in UC mice [298].

### 4.2. Regulation of Intestinal Microbiota

The homeostasis of the intestinal microbiota is critical to human intestinal health. In the gut of patients with IBD, an imbalance of the gut microbiota is commonly observed [299]. When the balance of intestinal flora is disrupted, the immune regulation and defense function of the intestine are compromised, causing damage to the intestinal mucosa and exacerbating IBD patients’ clinical symptoms [300]. A recent article summarized the changes in the microbiota of UC patients found in clinical studies. The results showed that in the gut of UC patients, the populations of *Firmicutes*, *Actinobacteria*, *Bacteroides*, *Streptococcus*, *Escherichia coli*, and *Fusobacterium nucleatum* increased and the populations of *Bacteroidetes*, *Bifidobacterium*, *Blautia*, *Coprococcus*, *Lachnospira*, *Lactobacillus*, *Roseburia*, *Ruminnococus*, *Akkermansia muciniphila*, *Bacteroides fragilis*, *Clostridium leptum*, *Eubacterium rectale*, *Faecalibacterium prausnitzi*, *Prevotella stercorea*, and *Ruminococcus gnavus* decreased [301]. Although it is not clear whether gut microbiota imbalance is a causative factor or a pathogenic consequence of IBD, many NPs have demonstrated that modulating the gut microbiota can improve clinical symptoms of experimental IBD [74,185,188,266].

The eight classes of NPs that we counted as having therapeutic effects on IBD all had different degrees of modulating effects on the intestinal microbiota. For example, the polyphenolic compound Salvianolic acid B could reduce the degree of inflammation in colitis by inhibiting the increase of *Bacteroides* and *Akkermansia* in the intestine of IBD mice [198]. Ginsenoside Rg1 significantly reversed the DSS-induced imbalance of intestinal microbiota in mice and attenuated acute UC symptoms through microbial tryptophan metabolism [169]. Juglone, a quinone, can increase the ratio of *Firmicutes* and *Bacteroidota*, increase the abundance of *Actinobacteriota*, and decrease the abundance of *Verrucomicrobiota* to alter the diversity and composition of intestinal microbes in UC mice [18].The coumarin-like compound daphnetin significantly increased the abundance of short-chain fatty acid-producing intestinal microbiota [253]. In addition, there are various natural polysaccharides that have a regulatory effect on the intestinal microbiota. It must be said that natural polysaccharides can provide a good growth environment for beneficial intestinal bacteria after digestion in the gastrointestinal tract, which is very beneficial for intestinal microorganisms [302]. Moreover, the fermentation of natural polysaccharides in the intestine can lower the pH value in the intestine, thereby inhibiting the growth of certain harmful microorganisms. The fermentation products of natural polysaccharides can also produce large amounts of short-chain fatty acids, which, in turn, can improve IBD through a variety of other pathways [303].

### 4.3. Protects the Intestinal Barrier Function

The altered permeability of intestinal mucosa is the primary manifestation of intestinal barrier dysfunction. The two main causes of intestinal barrier function damage are apoptosis of intestinal epithelial cells (IEC) and disruption of intercellular tight junctions (TJ) [304]. IEC is the barrier between the inner lumen and the external environment. Studies have shown that IEC apoptosis in the colon contributes to the development of chronic IBD [305]. IEC apoptosis is important in the pathogenesis of IBD. Excessive IEC apoptosis disrupts the intestinal defense system and impairs the function of the intestinal barrier [306]. Numerous studies have proved that NPs can inhibit IEC apoptosis through multiple pathways [188]. The main pathways involved are the death receptor-mediated pathway, mitochondria-dependent pathway, endoplasmic reticulum stress-mediated pathway, MAPK-mediated pathway, NF-κB-mediated pathway, and P13K/Akt-mediated pathway [307]. For example, biodegradation products of chitosan can prevent apoptosis in IEC by inhibiting NF-κB activation and decreasing TNF-α and IL-6 production [270]. TJ is a form of intercellular binding. Selective osmotic closure between adjacent IECs occurs due to the presence of TJ, which, in turn, forms an intestinal barrier to protect intestinal tissues. The cytoplasmic protein ZO (Zonula Occludens Proteins) family, transmembrane proteins (Tricellulin, Nectin, Occludin and Claudins), and cytoskeletal structures together constitute TJ [304]. Disruption of TJ leads to disruption of the intestinal immune system and inflammation, which is closely related to the development of IBD [308]. Alpinetin is a flavonoid isolated from *Alpinia katsumadai Hayata*. Alpinetin was found to improve intestinal barrier function in IBD mice by upregulating the expression of ocludin and zonula occludens-1 and downregulating the expression of claudin-2 [84]. Piperine is an alkaloid of natural origin. In an experimental IBD rat model, TNBS reduced the expression of claudin-1, ocludin, and zonula occludens-1 in colonic tissues, while Piperine significantly reversed the reduction in TJ expression brought about by TNBS [209]. These studies all confirmed that NPs can protect intestinal barrier function by inhibiting IEC apoptosis and reversing TJ disruption.

### 4.4. Reduces Inflammatory Response

Inflammation is a physiological phenomenon that occurs in the organism in response to injury, infection, and stress by the immune system [259]. The primary therapeutic effect of NPs in experimental IBD is improved colonic inflammatory response. Excessive inflammatory responses and pro-inflammatory factor overexpression aggravate colitis symptoms in IBD, and the inflammation is attributed to an over-response of immune cells in the organism, such as Th1 T-cell over-response or Th2 T-cell over-response [309]. The over-response of immune cells causes dramatic changes in cytokines, which include the pro-inflammatory cytokines IL-1, IL-2, IL-6, IL-12, IL-18, IFN-γ, and TNF-α and the anti-inflammatory cytokines IL-4, IL-5, IL-10, and IL-13. Many NPs modulate inflammatory cytokines through multiple pathways to attenuate the IBD inflammatory response. The flavonoid baicalin was reported to significantly reduce TNBS-induced inflammation in UC mice by decreasing the levels of IL-6, TNF-α, and IL-1β and increasing IL-10 [310]. Overexpression of TNF-α is thought to be one of the key factors in the pathogenesis of IBD, while TNF-α knockout mice were shown not to develop significant colitis [21]. In contrast, the natural terpenoid thymol could attenuate the inflammatory response in IBD mice by reducing the acetic acid-induced upregulation of pNF-κB p65 protein and significantly inhibiting the production of MPO and TNF-α in colonic tissues [150]. Zeaxanthin has an ameliorative effect on acetic acid-induced UC in rats. It was found that zeaxanthin ameliorated acetic acid-induced UC in rats by modulating the levels of pro-inflammatory cytokines (TNF-α, IFN-γ, IL-6, IL-1β, NF-κB) [153].

### 4.5. Improves Oxidative Stress

Numerous studies have confirmed that the development of IBD is closely related to the disruption of the antioxidant system. In the intestine of IBD patients, excessive levels of oxidative stress lead to lipid peroxidation, DNA damage, apoptosis, and inflammatory responses [311]. Excessive production of ROS, including superoxide anion radicals, hydrogen peroxide, and hydroxyl radicals, can disrupt the intestinal mucosal barrier by altering the concentration of oxidation-related enzymes and pro-inflammatory cytokines, triggering an inflammatory response and aggravating IBD [312]. In the IBD mouse model, the levels of nitric oxide (NO), myeloperoxidase (MPO), superoxide dismutase (SOD), malondialdehyde (MDA), and catalase (CAT) are altered in mouse colonic tissues, triggering an imbalance in intestinal function [313]. Many NPs have strong antioxidant activities, especially polyphenols and flavonoids. For example, the polyphenolic compound procyanidin B2 was found to have an ameliorative effect on the symptoms of IBD in experimental mice, and its potential mechanism of action is to inhibit oxidative stress in colonic tissues via Nrf2/ARE signaling, which, in turn, promotes the repair of intestinal damage [190]. The polyphenolic compound polydatin also reduced oxidative stress and apoptosis in the colon of IBD mice via the sonic hedgehog signaling pathway, thereby ameliorating DSS-induced acute UC [192]. Intervention with the flavonoid galangin in experimental IBD mice revealed that galangin treatment significantly inhibited the protein expression of p-NF-κB and p-Ikk-βα, COX-2, iNOS, and Nrf2 in the colonic tissues of UC mice and increased HO-1 levels by suppressing inflammation and oxidative stress [79]. In addition to this, some polysaccharides have been found to improve the level of oxidative stress in experimental IBD models. Examples are *Lycium barbarum* polysaccharides [211], *Dendrobium fimbriatum* polysaccharides [277], and *Dictyophora indusiata* polysaccharides [285].

### 4.6. Regulation of Immunity

In the development of IBD, changes in the immune system arise from abnormal responses of the innate and adaptive immune systems. In the peripheral blood of IBD patients and in vivo animal models, there is usually an increase in Th17 cells and a decrease in Treg cells [314]. Recent studies have shown that the immunometabolic processes in IBD mainly involve T cells, monocytes, macrophages, dendritic cells, and natural killer cells [315]. Immune cells produce inflammatory cytokines, which play an important role in regulating the inflammatory response process. As a result, targeting immune cell metabolism could be an effective way to treat IBD. Numerous studies have also demonstrated the ability of NPs to improve IBD symptoms by modulating immune metabolic processes in experimental IBD models. For example, nuciferine, an alkaloid, can improve DSS-induced UC in mice by regulating the differentiation of T cells and IgA+ B220+ B cells and restoring the balance of Th17/Treg and CD4+/CD8+ [215]. Similarly, celastrol improves Treg/Th1 and Treg/Th17 homeostasis in UC mice to maintain immune homeostasis in the gut [155]. *Ganoderma lucidum* polysaccharides have anti-inflammatory and immunomodulatory effects. In a DSS-induced IBD mouse model, Ganoderma lucidum polysaccharides could regulate the intestinal immune barrier function in mice by affecting the number of Th17 cells, B cells, NK cells, and NKT cells in lamina propria lymphocytes [284]. This suggested that targeting the metabolic processes of immune cells could be a novel approach to treating IBD.

### 4.7. Regulation of Key Signaling Pathways

Abnormal activation of signaling pathways triggers a dysregulated inflammatory response in patients with IBD [316]. Abundant studies have confirmed that different signaling pathways have important roles in the development and progression of IBD, and NPs can exert ameliorative and therapeutic effects on IBD through multiple signaling pathways.

#### 4.7.1. NF-κB Signaling Pathway

NF-κB is an important intracellular nuclear transcription factor. It is involved in the inflammatory response of the body, immune response, and can regulate apoptosis and stress response. Over-activation of NF-κB is closely associated with many inflammation-related diseases in humans [317]. In the chemical agent-induced IBD model, the NF-κB signaling pathway is activated and plays an important role in the course of IBD [318]. And the inhibition of NF-κB signaling pathway by drugs may become a possible way to treat IBD. Most experimental results on the improvement of IBD by NPs have found their modulatory effects on NF-κB signaling pathway. In this paper, we summarized eight classes of compounds that could all improve the symptoms of experimental IBD through the NF-κB signaling pathway. For example, ganoderic acid C1, isolated from *Ganoderma lucidum*, downregulated the NF-κB signaling pathway in colon tissue, thereby significantly reducing the production of TNF-α, IFN-γ, and IL-17A in colon tissue of CD patients [143]. Isobavachalcone, a natural flavonoid compound, significantly improved clinical signs in mice with experimental IBD. The potential mechanism was found to improve colitis in mice by inhibiting NF-κB p65 [105]. Imperatorin, a naturally occurring coumarin, inhibits NF-κB nuclear translocation and downregulates pro-inflammatory gene expression in UC mice. In vivo experiments demonstrated that imperatorin ameliorated DSS-induced colitis by inhibiting NF-κB signaling [257].

#### 4.7.2. MAPK Signaling Pathway

The MAPK signaling pathway is one of the classical inflammatory signaling pathways that can be activated in response to external stimuli. When the MAPK signaling pathway is activated, it mainly involves changes in extracellular signal-regulated kinases 1 and 2 (ERK1/2), C-Junn-terminal kinases 1,2,3 (JNK1/2/3), and p38 [319]. MAPK is an upstream signaling molecule of NF-κB, which also plays an important role in inflammatory diseases. It was found that the expression of MAPK signaling pathway-related proteins was significantly increased in the colonic tissues of a DSS-induced IBD mouse model, which indicated the activation of the MAPK signaling pathway in the IBD mouse model [320]. Similarly, some NPs can improve IBD by inhibiting the activation of the MAPK signaling pathway. In DSS-induced UC in mice, the MAPK signaling pathway is activated, and naringin can protect mice from DSS injury by inhibiting the activation of the MAPK signaling pathway [113]. Osthole, a natural coumarin, can exert anti-inflammatory effects in an experimental IBD mouse model by blocking the activation of NF-κB and MAPK/p38 pathways, thereby effectively alleviating clinical symptoms in UC mice [249]. Among the 170 NPs we pooled, compounds **42**, **73**, **74**, **92**, **106**, **114**, **134**, **142**, and **169** all exerted ameliorative effects on IBD via the MAPK signaling pathway.

#### 4.7.3. JAK/STAT Signaling Pathway

The JAK/STAT signaling pathway is an important downstream pathway of cytokines, including JAK1, JAK2, JAK3, TYK2, and STAT proteins. The JAK/STAT signaling pathway has an important role in regulating mucosal injury, inflammation, and immune regulation [321]. Some researchers investigated the anti-inflammatory effects of cinnamaldehyde and hesperetin by constructing a TNBS-induced rat IBD model. The results showed that cinnamaldehyde and hesperetin significantly improved the macroscopic pathology of rats and significantly reduced oxidative stress in the colon. In addition, they significantly decreased the expression of p-JAK2 and p-STAT3, and improved TNBS-induced UC by regulating the JAk2/STAT3/SOCS3 signaling pathway [92]. In addition, (+)-Borneol, a natural terpenoid, promotes M2 macrophage polarization via the JAK2-STAT3 signaling pathway, thereby enhancing the efficacy of edaravone against DSS-induced colitis [139]. *Chrysanthemum* polysaccharides can improve the pathological condition of UC rats. The investigators explored the mechanism of action using a TNBS-induced rat IBD model. The results showed that *Chrysanthemum* polysaccharides could improve the pathological condition of UC rats by modulating the NF-κ B/TLR4 and IL-6/JAK2/STAT3 signaling pathways [273].

#### 4.7.4. PI3K and TLRs

Phosphatidylinositol-3-kinase (PI3K) can be involved in the regulation of cell surface receptor signaling and can regulate the activation, growth, and proliferation of leukocytes. It was found that resveratrol could attenuate acute UC in mice by inhibiting the activation of the PI3K/AKT pathway in a DSS-induced IBD mouse model [183]. Another study found that oxymatrine, a natural alkaloid, was also able to improve DSS-induced UC by inhibiting the PI3K/AKT signaling pathway [223]. Aloperine was also found to ameliorate the inflammatory response in the colon of UC mice by inhibiting PI3K/Akt/mTOR signaling in a PP2A-dependent manner [227]. Toll-like receptors (TLRs) are an important class of protein molecules involved in nonspecific immunity, and TLRs can recognize abnormal molecules and activate the body to produce an immune response. TLRs play an important role in the homeostasis of the intestinal mucosa. In contrast, TLR expression is reduced in experimental IBD models, especially TLR4 [273]. A variety of NPs are involved in changes in the TLRs pathway. For example, eriodictyol, a natural flavonoid, can inhibit UC progression by modulating TLR4/NF-κB pathway activation in a TNBS-induced experimental IBD rat model [96]. Betulin attenuated acetic acid-induced UC in rats by downregulating the TLR4/NF-kB axis, which, in turn, reduced TNF-α and IL-6 levels and caspase-3 and caspase-8 expression in colonic tissues [152]. Among the NPs we summarized, the ameliorative effects of compounds **9**, **10**, **15**, **17**, **66**, **82**, **108**, **120**, **127**, **144**, **152**, and **169** on IBD all involved TLRs pathways.

#### 4.7.5. NLRP3 Inflammatory Vesicles

Inflammatory vesicles are an important component of the innate defense of the body and play an important role in the development and progression of IBD [322]. Among the many pattern recognition receptors, NLRP3 inflammatory vesicles are most closely associated with IBD and are the inflammatory vesicles that have received the most attention and study by researchers [323]. NLRP3 inflammatory vesicles, an innate immune supramolecular assembler, are upregulated in patients with IBD and in experimental animal models and are continuously activated by multiple stimuli during the course of IBD [324]. Many NPs can alleviate the clinical symptoms of experimental IBD models by inhibiting the activation of NLRP3 inflammatory vesicles, thereby improving and treating IBD. For example, oroxindin, a natural flavonoid, can attenuate the inflammatory response in the colon of an experimental IBD mouse model by inhibiting the formation and activation of NLRP3 inflammatory vesicles [82]. Similarly, the natural flavonoid cardamonin was able to alleviate UC in mice by activating the AhR/Nrf2/NQO1 pathway and inhibiting the activation of NLRP3 inflammatory vesicles [115]. The natural terpenoid Asiatic acid was also found to improve DSS-induced UC in experimental mice by inhibiting mitochondria-mediated activation of NLRP3 inflammatory vesicles [133]. In this paper, compounds **3**, **32**, **33**, **46**, **70**, **81**, **93**, **126**, and **128** showed inhibition of NLRP3 inflammatory vesicles in an experimental IBD model.

#### 4.7.6. PPARγ

Peroxisome proliferator-activated receptors (PPARs), a nuclear receptor highly expressed in the colon, are involved in the control of gene expression in various physiological processes and play a key role in the development of inflammation [325,326]. There are three different isoforms of PPARs, namely PPARα, PPARβ, and PPARγ. Among them, PPARγ plays a key role in the process of IBD and is used as a target for the development and treatment of IBD drugs [326]. Recent studies have shown that some NPs can exert ameliorative effects on IBD by activating or modulating PPARγ. For example, Thymoquinone, a plant-derived NPs, significantly increased the expression of PPAR-γ mRNA and PPAR-γ protein in the intestine of experimental IBD mice and enhanced the activity of PPAR-γ promoter [327]. Further, α-bisabolol, a natural monocyclic sesquiterpene enol, was found to be a specific stimulator of PPARγ. In the experiment, α-bisabolol was found to enhance the expression of PPARγ transcription factor in the colonic epithelium of mice with experimental IBD, but the expression of PPARα and PPARβ was not altered. The investigators asserted that α-bisabolol could reduce colonic inflammation by stimulating PPARγ expression [328]. Furthermore, emodin also increased the expression of PPAR-γ in the colon of UC mice and had a synergistic effect with baicalin [123]. Among the 170 NPs we summarized, compounds **9**, **30**, **51**, **57**, **71**, **94**, **105**, and **144** were also found to increase the expression of PPAR-γ.

## 5. Conclusions and Prospect

In this paper, we reviewed the pathogenesis of IBD as well as the current therapeutic approaches and therapeutic agents. IBD as a chronic, non-specific inflammatory disease of the intestinal tract is associated with genetics, environment, intestinal microbes, hygiene, diet, sleep, mental health, smoking, antibiotic use, and post-surgical complications. Current therapeutic agents and treatments include amino salicylic acid preparations, corticosteroids, immunomodulators, biologics, stem cell transplantation, fecal microbiota transplantation, helminth therapy, and surgery. However, it is clear that these drugs and methods do not meet the treatment needs of all patients.

The biological activity of NPs has generated great interest among researchers in many fields, such as the pharmaceutical, health food, and cosmetic industries [329]. NPs are considered to be a promising source of drugs for the prevention and improvement of IBD due to their low side effects, high safety profile, high activity, and multiple ameliorative and therapeutic effects on IBD [301]. Therefore, in this paper, we summarized eight categories of 170 NPs with therapeutic and ameliorative effects on experimental IBD and analyzed the potential mechanisms of their effects. It was found that NPs could exert therapeutic and ameliorative effects on experimental IBD by improving the pathological symptoms of IBD, regulating intestinal microbiota, protecting intestinal barrier function, reducing inflammatory response, improving oxidative stress, and regulating immunity, among which the main signaling pathways involved were NF-κB, MAPK, JAK/STAT, PI3K, TLRs, NLRP3 inflammatory vesicles, and PPARγ, etc.

Although important research advances and key findings have been made regarding the therapeutic effects of NPs on IBD, there are still some issues that need to be further explored and addressed. First, most studies were limited to cellular level and in vivo studies in animals. Although animal models of IBD cannot fully mimic human clinical outcomes, researchers can play an important role in understanding human IBD pathology by selecting an appropriate IBD model with due consideration of caveats. Having said that, experimental IBD models are a viable option for determining the underlying mechanisms causing IBD [330]. This paper also mainly explored the role of NPs in experimental IBD models, and further in-depth validation is needed for human experiments and clinical trial level. Secondly, the active components and structures of some NPs need to be further elaborated, such as the amino acid structures of protein peptides and the composition of natural polysaccharide monosaccharides, which need to be further purified, characterized, and modified to improve their activities. Moreover, due to the limitation of research methods, the mechanism of action of some NPs has not been fully elucidated, and further studies are needed to elucidate their mechanism of action in detail and provide new and more therapeutic targets for the treatment of IBD. In addition, although some NPs have been used as conventional drugs for the treatment of IBD, the systematic evaluation of their toxicity and safety is insufficient, and more attention needs to be paid to the potential toxicity and adverse effects of NPs. From the perspective of NPs as drugs for the treatment of IBD, the pharmacokinetic and pharmacodynamic studies are still significantly inadequate and need to be further explored to lay the foundation for subsequent toxicological and safety evaluations [331]. Finally, the combination of different NPs exhibited better therapeutic effects in IBD, so studies on drug combination aspects are also necessary.

Moreover, drug delivery and targeted therapies for NPs remain the focus of future exploration. While conventional oral NPs have low bioavailability and limited potential physiological activity, nanoparticles can significantly improve the bioavailability of NPs during their delivery. It has been demonstrated that the delivery of NPs in the form of nano formulations and nanoparticles to the site of colonic inflammation can increase the effective concentration of the drug at the site of inflammation, thereby significantly improving the efficacy of NPs in the treatment of IBD and reducing the complications associated with conventional drug delivery [332,333]. The wide application of nanotechnology in the pharmaceutical field offers great possibilities for the use of NPs for the targeted treatment of IBD. More new and untested nano-agents of NPs should be vigorously researched and developed in the future. Nanorobots might also be used as delivery vehicles for NPs. In addition, there is an urgent need for more clinical trials of NP nano-agents to validate their therapeutic efficacy.

Addressing the above issues is a huge challenge but made possible by technological developments and scientific research in many aspects of bioscience, drug modification, nanotechnology, and clinical research. Advanced interdisciplinary and cross-disciplinary research will collectively contribute to the therapeutic effects of NPs for IBD.

In conclusion, we expect that this review will provide a systematic and credible summary in order to provide useful information for researchers to understand the therapeutic and ameliorative effects of NPs on IBD, which, in turn, will provide a theoretical basis for the development and application of NPs in drugs and functional foods. We also expect more researchers and physicians to pay more attention to the benefits of NPs in IBD and actively conduct more related studies.

## Figures and Tables

**Table 1:** In vivo therapeutic effects of flavonoids on experimental IBD.

No.	Name	Type	Origin	Optimal Doses (/kg Body Weight)	Model	Potential Mechanism	References
1	Galangin	Hydroxyflavonol	*Alpinia conchigera*	40 mg	DSS-induced ulcerative colitis in BALB/c mice	Inhibit inflammation and oxidative stress.	[79]
				40 mg	DSS-induced colitis in Swiss albino mice	Downregulation of toll-like receptor 4 expression, inhibition of NF-κB p65 activation, and reduction of inflammatory factor levels.	[80]
2	Pinocembrin	Dihydroxyflavanone	*Prunus leveilleana*	10 mg	DSS-induced rats ulcerative colitis model	Improves inflammation levels, intestinal barrier function, and regulates the intestinal microbiota.	[81]
3	Oroxindin	Flavonoid	*Scutellaria discolor*	12.5 mg	DSS-induced rats ulcerative colitis model	Attenuates the inflammatory response by inhibiting the formation and activation of NLRP3 inflammatory vesicles.	[82]
4	Myricetin	Hexahydroxyflavone	*Ficus auriculata*	80 mg	DSS-induced C57BL/6 mice ulcerative colitis model	Significantly increased the levels of IL-10, transforming growth factor b and the proportion of regulatory T cells.	[83]
5	Alpinetin	Flavonoid	*Alpinia blepharocalyx*	100 mg	DSS-induced mice ulcerative colitis model	Reduces intestinal inflammation and oxidative stress dose-dependently associated with and regulates the expression of tight junctions between cells in UC mice.	[84]
				50 mg	DSS-induced C57BL/6 mice ulcerative colitis model	Helps to eliminate chemically induced IBD by activating PXR (a ligand of human pregnane X receptor).	[85]
6	Pectolinarigenin	Dimethoxyflavone	*Eupatorium cannabinum*	10 mg	DSS-induced C57BL/6 mice ulcerative colitis model	Dose-dependent reduction of DSS-induced colonic inflammation through modulation of NF-κB/Nrf2 signaling pathway and enhancement of myeloperoxidase (peroxisome) activity and redox regulators.	[86]
7	Casticin	Tetramethoxyflavone	Viticis Fructus	/	DSS-induced C57BL/6 mice ulcerative colitis model	Alleviation of DSS-induced UC by increasing the expression of the antioxidant enzymes peroxidase 3 and MnSOD and reduction of pro-inflammatory chemokine production by inhibiting AKT signaling.	[87]
8	Hyperoside	Tetramethoxyflavone	*Lotus ucrainicus*	120 mg	DSS-induced acute colitis in mice	Reduction of colonic inflammation and apoptosis through activation of the Nrf2 signaling pathway.	[88]
9	Phloretin	Dihydrochalcone	*Malus doumeri*	100 mg	DSS-induced C57BL/6 mice ulcerative colitis model	Inhibits inflammatory responses by regulating nuclear factor-κB (NF-κB), toll-like receptor 4 (TLR4), and peroxisome proliferator-activated receptor γ (PPARγ) pathways.	[89]
10	Wogonin	Dihydrochalcone	*Scutellaria likiangensis*	10 mg	DSS-induced acute colitis in C57BL/6 mice	Significantly reduced the intestinal inflammatory response in IBD mice by increasing the expression of IL-10.	[90]
				50 mg	DSS-induced acute colitis in BALB/c mice	Regulates the Nrf2 signaling pathway and reduces TLR-4/NF-κB triggering.	[91]
11	Cinnamaldehyde	Flavonoid	Cinnamon trees	90 mg	TNBS-induced ulcerative colitis in rats	Inhibition of TNBS-induced UC through antioxidant and anti-inflammatory properties and modulation of the JAk2/STAT3/SOCS3 pathway.	[92]
				10 mg	DSS-induced acute colitis in BALB/c mice	Inhibition of Th17 cell differentiation via sphingosine-1-phosphate receptor 2.	[93]
12	Hesperetin	Flavonoid	*Brassica oleracea var. sabauda*	100 mg	TNBS-induced ulcerative colitis in rats	Inhibition of TNBS-induced UC through antioxidant and anti-inflammatory properties and modulation of the JAk2/STAT3/SOCS3 pathway.	[92]
13	Tiliroside	Glycosyloxyflavone	*Galphimia gracilis*	50 mg	DSS-induced colitis model; TNBS-induced colitis model	Restoration of M1/M2 macrophage homeostasis through the HIF-1a/glycolytic pathway, resulting in improved UC.	[94]
14	Didymin	Flavonoid	*Citrus latipes*	4 mg	DSS-induced acute colitis in C57BL/6 mice; DSS-induced chronic colitis in C57BL/6 mice	Didymin converts m1-like macrophages to m2-like macrophages and ameliorates UC through fatty acid oxidation.	[95]
15	Eriodictyol	Flavanone	*Prunus campanulata*	50 mg	TNBS-induced animal model of enteritis in Wistar rats	Attenuation of TNBS-induced UC by inhibition of TLR4/NF-kB signaling pathway in rats.	[96]
				50 mg	DSS-induced acute colitis in C57BL/6 mice	Eriodictyol attenuates DSS-induced UC in mice by regulating the sonic hedgehog signaling pathway.	[97]
16	Tricin	Trihydroxyflavone	*Carex fraseriana*	150 mg	DSS-induced acute colitis in BALB/c mice	Improves colonic inflammation and regulates intestinal microbiota.	[98]
17	Pinocembrin	Dihydroxyflavanone	*Prunus leveilleana*	50 mg	DSS-induced acute colitis in C57BL/6 mice	By modulating the intestinal flora, inhibiting the excessive activation of TLR4/MD2/NF-κB signaling pathway, and improving the intestinal barrier, thereby reducing DSS-induced colitis in mice.	[99]
18	Astragalin	Trihydroxyflavone	*Salix atrocinerea*	100 mg	DSS-induced acute colitis in C57BL/6J mice	Attenuation of DSS-induced acute UC by attenuating intestinal microbiota dysbiosis and inhibiting NF-κB activation in mice.	[100]
19	Kaempferol	Tetrahydroxyflavone	*Lotus ucrainicus*	50 mg	DSS-induced acute colitis in C57BL/6J mice	Reduction of experimental colitis in mice by restoring the intestinal microbiota and inhibiting the LPS-TLR4-NF-kB axis.	[101]
20	Acacetin	Dihydroxyflavanone	*Verbascum lychnitis*	50 mg	DSS-induced acute colitis in C57BL/6 mice	Improvement of experimental colitis in mice by inhibiting the inflammatory response of macrophages and modulating the composition of the intestinal microbiota.	[102]
21	Genistein	Isoflavone	*Felmingia vestita*	10 mg	DSS-induced acute colitis in C57BL/6 mice	Polarization of M1 to M2 macrophages and a reduction in systemic cytokines partially reduce the symptoms of colitis.	[103]
22	Oroxylin A	Dihydroxyflavanone	*Scutellaria likiangensis*	50 mg	DSS-induced acute colitis in C57BL/6J mice	Maintains the colonic mucus barrier and regulates the intestinal microbiota.	[104]
23	Isobavachalcone	Trans-chalcone	*Broussonetia papyrifera*	50 mg	DSS-induced acute colitis in C57BL/6 mice	Amelioration of colitis in mice by inhibition of the NF-κB p65 pathway.	[105]
24	Naringenin	Flavanone	*Elaeodendron croceum*	50 mg	DSS-induced acute colitis in C57BL/6 mice	Protective effects on experimental colitis through inhibition of toll-like receptor 4/NF-κB signaling.	[106]
25	Nobiletin	Methoxyflavone	*Citrus tankan*	50 mg	Ethanol-induced colitis in BALB/c mice	Reduced inflammatory signs and markers of colitis and deposition and expression of fibrotic collagen in mice.	[107]
26	Luteolin	Tetrahydroxyflavone	*Verbascum lychnitis*	/	DSS-induced acute colitis in C57BL/6 mice	Inhibits the elevation of 5-hydroxytryptamine.	[108]
				50 mg	DSS-induced acute colitis in C57BL/6 mice	Amelioration of colitis in mice by activating the Nrf2 signaling pathway.	[109]
27	Taxifolin	Dihydroflavonol	*Salix atrocinerea*	10 mg	DSS-induced acute colitis in C57BL/6 mice	Alleviation of UC by acting on the gut microbiota to produce butyric acid.	[110]
28	Icariin	Glycosyloxyflavone	*Epimedium pubescens*	10 mg	DSS-induced acute colitis in C57BL/6 mice	Suppression of intestinal inflammation in UC mice through modulation of intestinal flora abundance and regulation of p-p65/p65 molecules.	[111]
29	Baicalein	Trihydroxyflavone	*Scutellaria baicalensis*	40 mg	DSS-induced acute colitis in C57BL/6 mice	Improvement of the intestinal epithelial barrier through the AhR/IL-22 pathway in innate lymphoid cells.	[112]
30	Naringin	Dihydroxyflavanone	*Citrus latipes*	100 mg	DSS-induced acute colitis in C57BL/6 mice	Inhibition of NF-κB and MAPK activation and regulation of the PPARγ pathway.	[113]
31	Puerarin	Dydroxyisoflavone	*Neustanthus phaseoloides*	50 mg	DSS-induced acute colitis in BALB/c mice	Regulation of Nrf2 and antioxidant enzyme expression.	[114]
32	Cardamonin	Chalcone	*Amomum subulatum*	60 mg	DSS-induced acute colitis in C57BL/6 mice; TNBS-induced colitis in BALB/c mice	Inhibition of NLRP3 inflammatory vesicle activation via the AhR/Nrf2/NQO1 pathway.	[115]
33	Curcumin	Diketone	*Curcuma longa*	20 mg	DSS-induced acute colitis in C57BL/6 mice	Inhibition of NLRP3 inflammasome activation and IL-1β production.	[116]
				100 mg	DSS-induced ulcerative colitis complicating diabetes in C57BLKS/J mice	Restores Th17/Treg homeostasis and improves the composition of the intestinal flora.	[117]
34	Quercetin	Pentahydroxyflavone	*Quercus*	10 mg	DSS-induced acute colitis in C57BL/6 mice	Inhibition of colitis by induction of anti-inflammatory effects of macrophages and alteration of intestinal flora.	[118]
35	Daidzein	Hydroxyisoflavone	*Pericopsis elata*	10 mg	DSS-induced acute colitis in BALB/c mice	Regulation of NF-κB signaling pathway.	[119]
36	Cyanidin	Flavonoid	*Salix atrocinerea*	64.5 mg	TNBS-induced colitis model in BALB/c mice	Protects the intestinal barrier as well as inhibits the secretion of inflammatory cytokines.	[120]
37	Cyanidin-3-O-Glucoside	Flavonoid	*Ipomoea batatas*	96.8 mg	TNBS-induced colitis model in BALB/c mice	Protects the intestinal barrier as well as inhibits the secretion of inflammatory cytokines.	[120]
38	Eriocitrin	Trihydroxyflavanone	*Citrus latipes*	30 mg	DSS-induced acute colitis in C57BL/6J mice	Reduced MPO content, MMP-9, and NFκB activation. Inhibited the production of pro-inflammatory cytokines and the expression of iNOS and COX-2.	[121]
39	Hesperidin methylchalcone	Flavonoid	*Myrtus communis*	30 mg	Acetic acid-induced colitis in Swiss and LysM-eGFP mice	Increases antioxidant response and reduces inflammation.	[122]
40	Baicalin	Dihydroxyflavanone	*Scutellaria amoena*	100 mg	DSS-induced acute colitis in C57BL/6J mice	Decreased the expression of CD14 and inhibited NF-κB activity.	[123]
41	kurarinone	Flavonoid	*Sophora flavescens*	200 mg	DSS-induced acute colitis in C57BL/6J mice	Improvement of UC through regulation of Th17/Treg cell homeostasis.	[124]
42	A-type proanthocyanidin	Flavonoid	*Geranium niveum*	/	DSS-induced acute colitis in BALB/c mice	Regulation of intestinal microbiota and colonic metabolism.	[125]
43	Linarin	Glucoside	*Chrysanthemum indicum*	50 mg	DSS-induced acute colitis in C57BL/6J mice	Improves intestinal barrier, inhibits inflammatory response, and regulates intestinal microbiota.	[126]
44	Vitexin	Trihydroxyflavone	*Itea omeiensis*	/	DSS-induced acute colitis in mice	Reduced inflammation, intestinal barrier dysfunction, and intestinal flora dysbiosis in mice with colitis.	[127]
45	Licoflavone B	Isoprene flavonoid	*Glycyrrhiza glabra*	120 mg	DSS-induced colitis in C57BL/6 mice	Rebuilding the intestinal barrier and regulating intestinal flora.	[128]
46	Trifolirhizin	Isoflavone	*Sophora flavescens*	50 mg	DSS-induced colitis in C57BL/6 mice	Regulation of Th17/Treg cell homeostasis and inflammation in UC mice through inhibition of TXNIP-mediated NLRP3 inflammatory vesicle activation.	[129]
47	Calycosin	O-methylated isoflavone	*Astragalus membranaceus*	50 mg	DSS-induced acute colitis in BALB/c mice	Significantly inhibited NF-κB pathway and JNK phosphorylation.	[130]
48	Apigenin	Trihydroxyflavone	*Cordia dichotoma*	/	DSS-induced chronic colitis in C57BL/6 mice	Anti-inflammatory effects through inhibition of classical and non-classical inflammatory vesicle signaling pathways.	[131]

“/” indicates that the reference is not mentioned or is unclear.

**Table 2:** In vivo therapeutic effects of terpenoids on experimental IBD.

No.	Name	Type	Origin	Optimal Doses (/kg Body Weight) or Concentrations	Model	Potential Mechanism	References
49	Dihydrotanshinone I	Diterpenoid	*Salvia miltiorrhiza*	25 mg	DSS-induced acute colitis in C57BL/6J mice	Attenuation of DSS-induced UC in mice by inhibition of pro-inflammatory mediators and modulation of the RIPs-MLKL-caspase-8 axis.	[136]
50	Plumericin	Terpene lactone	*Himatanthus drasticus*	3 mg	DNBS-induced colitis in CD1 mice	Reduces inflammation and oxidative stress.	[135]
51	β-caryophyllene	Bicyclic sesquiterpene	*Syzygium aromaticum*	50 mg	DSS- and oxazolone-induced acute colitis in CD1 mice	Anti-inflammatory effects via CB2 and PPARγ pathways.	[137]
52	α-Amyrin	Pentacyclic triterpenoid	*Ficus pertusa*	10 mg	DSS-induced acute colitis in CD1 mice	Reduces leukocyte influx into the colon; inhibits the production of pro-inflammatory cytokines; decreases mRNA expression of colonic adhesion molecules.	[138]
53	β-Amyrin						
54	(+)-Borneol	Bicyclic monoterpene	*Blumea balsamifera*	3 mg	DSS-induced acute colitis in C57BL/6 mice	Promoting M2 macrophage polarization through the JAK2-STAT3 signaling pathway.	[139]
55	β-Carotene	Tetraterpene carotenoid	*Dunaliella salina*	20 mg	DSS-induced acute colitis in Swiss Albino mice	Reduces inflammation, oxidative stress, fibrosis and DNA damage in the colon.	[140]
56	Carvacrol	Phenolic monoterpene	*Origanum vulgare*	100 mg	Acetic acid-induced colitis in C57BL/6 mice	Reduces inflammation, injurious nociceptive, and oxidative damage.	[134]
57	Geraniol	Monoterpenoid	*Cinnamomum tenuipilum*	120 mg	DSS-induced acute colitis in C57BL/6 mice	Reduction of ecological disorders and systemic inflammation.	[141]
				250 mg	TNBS-induced colitis in Wistar rats	Reduction of colitis through Wnt/β-catenin, p38MAPK, NFκB, and PPARγ signaling pathways.	[142]
58	Ganoderic acid C1	Triterpenoid	*Ganoderma lucidum*	40 μg/mL	Lamina Propria Mononuclear Cells	Downregulation of NF-κB signaling.	[143]
59	D-Limonene	Cyclic monoterpene	*Vitis rotundifolia*	10 mg	TNBS-induced colitis in Wistar HsdBrlHan rats	Inhibits the inflammatory response.	[144]
				100 mg	TNBS-induced colitis in Sprague–Dawley rats	Exhibits anti-inflammatory and antioxidant properties through modulation of iNOS, COX-2, PGE2, and ERK signaling pathways.	[145]
60	Menthol	Cyclic monoterpene	*Chaerophyllum macrospermum*	80 mg	Acetic acid-induced colitis in Wistar rats	Significantly reduces inflammation.	[146]
61	Nerol	Monoterpenoid alcohol	*Citrus aurantium*	300 mg	Oxazolone-induced colitis in BALB/c mice	Improves the pathological features of colitis, protects the stomach from damage, and has immunomodulatory effects.	[147]
62	Oleanolic Acid	Pentacyclic triterpenoid	*Ophiopogon japonicus*	10 mg	DSS-induced acute colitis in C57BL/6 mice	Restoration of Th17/Treg cell homeostasis and inhibition of NF-κB signaling pathway.	[148]
62	Perillaldehyde	Monoterpene	*Perilla frutescens*	100 mg	DSS-induced acute colitis in C57BL/6 mice	Improvement of intestinal inflammation through JNK-mediated cytokine regulation.	[149]
64	Thymol	Monoterpene	*Xylopia aromatica*	100 mg	Acetic acid-induced colitis in Wistar rats	Inhibition of NF-kB signaling pathway to reduce inflammatory response.	[150]
65	Alantolactone	Sesquiterpene lactone	*Eupatorium cannabinum*	50 mg	DSS-induced acute colitis in C57BL/6 mice	Inhibition of NF-κB inflammatory signaling mediated by PXR.	[151]
66	Betulin	Pentacyclic triterpenoid	*Diospyros morrisiana*	8 mg	Acetic acid-induced colitis in Sprague–Dawley rats	Inhibition of colonic apoptosis by reducing colonic caspase-3 and caspase-8 expression; potential mechanisms include downregulation of TLR4/NF-κB and subsequent downstream signaling pathways.	[152]
67	Zeaxanthin	Carotenoid	*Bangia fuscopurpurea*	50 mg	Acetic acid-induced colitis in Sprague Dawley rats	Regulation of pro-inflammatory cytokines and oxidative stress.	[153]
68	D-Carvone	Terpenoid	*Carum carvi*	40 mg	DSS-induced acute colitis in BALB/c mice	Inhibition of COX-2 and TNF-α.	[154]
69	Celastrol	Triterpenoid	*Tripterygium wilfordii*	1 mg	DSS-induced acute colitis in BALB/c mice	Improves Treg/Th1 and Treg/Th17 balance to maintain colonic immune homeostasis; regulates intestinal microbiota.	[155]
70	Asiatic acid	Triterpenoid	*Centella asiatica*	30 mg	DSS-induced acute colitis in C57BL/6 mice	Inhibits mitochondria-mediated activation of NLRP3 inflammatory vesicles.	[133]
71	Madecassic acid	Triterpenoid	*Centella asiatica*	25 mg	DSS-induced acute colitis in C57BL/6 mice	Inhibition of γδT17 cell activation via PPARγ-PTEN/Akt/GSK3β/NFAT pathway.	[156]
72	Nerolidol	Sesquiterpene alcohol	*Brassavola nodosa*	150 mg	DSS-induced acute colitis in C57BL/6J mice	Reduce colonic inflammation by exerting its antioxidant and anti-inflammatory activities.	[157]
73	β-Myrcene	Monoterpene	*Teucrium montanum*	100 mg	DSS-induced acute colitis in C57BL/6J mice	Inhibition of MAPK and NF-κB pathways.	[158]

**Table 3:** In vivo therapeutic effects of glycosides on experimental IBD.

No.	Name	Type	Origin	Optimal Doses (/kg Body Weight)	Model	Potential Mechanism	References
74	Dioscin	Steroidal saponin	*Ophiopogon intermedius*	160 mg	DSS-induced acute colitis in BALB/c mice	Regulates the polarization of macrophages.	[172]
75	Pelargonidin 3-glucoside	Anthocyanidin glycoside	*Lonicera caerulea*	8 mg	DSS-induced chronic IBD in rats	Reduces inflammation and reduces IBD symptoms.	[173]
76	2,3,5,4′-Tetrahydroxystilbene-2-O-β-D-glucoside	Glucoside	*Polygonum multiflorum*	100 mg	DSS-induced acute colitis in BALB/c mice	Reduces inflammation and regulates the intestinal microbiota.	[174]
77	Ginsenoside Rb1	Ginsenoside	*Panax vietnamensis*	40 mg	DSS-induced acute colitis in C57BL/6 mice	Attenuation of mouse colitis by activation of the endoplasmic reticulum resident E3 ubiquitin ligase Hrd1 signaling pathway.	[16]
78	Ginsenoside Rd	Ginsenoside	*Panax vietnamensis*	20 mg	Sprague–Dawley rats injected with indomethacin	Stimulates the proliferation and differentiation of endogenous intestinal stem cells and restores intestinal function.	[168]
79	Ginsenoside Rg1	Ginsenoside	*Panax vietnamensis*	200 mg	DSS-induced acute colitis in C57BL/6 mice	Alleviation of acute UC by modulating gut microbiota and microbial tryptophan metabolism.	[169]
80	Ginsenoside Rh2	Ginsenoside	*Panax vietnamensis*	50 mg	DSS-induced acute colitis in C57BL/6J mice	Mitigation of UC by regulating STAT3/miR-214 signaling pathway.	[170]
81	Ginsenoside Rk3	Ginsenoside	*Panax vietnamensis*	40 mg	DSS-induced acute colitis in C57BL/6 mice	Protection of the colonic barrier and inhibition of NLRP3 inflammatory vesicles.	[171]
82	Paeoniflorin	Terpene glycoside	*Paeonia*	50 mg	DSS-induced acute colitis in C57BL/6 mice	Inhibition of NF-κB and MAPK pathway activation by reducing TLR4 expression.	[175]
				20 mg	DSS-induced acute colitis in C57BL/6 mice	Inhibition of inflammatory response and eosinophil infiltration.	[161]
83	Salidroside	Glycoside	*Salix atrocinerea*	40 mg	DSS-induced acute colitis in C57BL/6 mice	Protection of mice with colitis by activation of the SIRT1/FoxOs pathway, which is associated with oxidative stress and apoptosis in colonic tissues.	[162]
84	Anemoside B4	Saponin	*Pulsatilla chinensis*	100 mg	DSS-induced acute colitis in C57BL/6 mice	Regulation of inflammatory response, colonic transcriptome, and intestinal microbiota.	[176]
85	Wogonoside	Flavonoid glycoside	*Scutellaria baicalensis*	50 mg	DSS-induced acute colitis in C57BL/6 mice	Improving intestinal epithelial barrier function through the MLCK/pMLC2 pathway alleviates colitis.	[163]
86	Hesperidin	Flavanone glycoside	*Citrus aurantium*	40 mg	DSS-induced acute colitis in C57BL/6 mice	Prevents intestinal inflammation by restoring intestinal barrier function and upregulating Treg cells.	[164]

**Table 4:** In vivo therapeutic effects of polyphenolic compounds on experimental IBD.

No.	Name	Type	Origin	Optimal Doses (/kg Body Weight)	Model	Potential Mechanism	References
87	Epigallocatechin-3-gallate	Catechin	*Limoniastrum guyonianum*	50 mg	DSS-induced acute colitis in C57BL/6J mice	Improves intestinal epithelial homeostasis and regulates intestinal microbiota.	[188]
88	Gallic acid	Trihydroxybenzoic acid	*Visnea mocanera*	10 mg	DSS-induced acute colitis in BALB/c mice	Downregulation of IL-21 and IL-23 expression levels. Activation of enzymatic antioxidants via the Nrf2 pathway to provide cryoprotection.	[189]
89	Procyanidin B2	Polyphenol	*Begonia fagifolia*	30 mg	DSS-induced acute colitis in C57/BL6 mice	Inhibition of oxidative stress through the Nrf2/ARE signaling pathway, which in turn promotes intestinal damage repair.	[190]
90	Procyanidin A1	Polyphenol	*Tainia latifolia*	10 mg	DSS-induced acute colitis in BALB/c mice	Regulation of AMPK/mTOR/p70S6K-mediated autophagy.	[191]
91	Polydatin	Polyphenol	*Vitis rupestris*	45 mg	DSS-induced acute colitis in C57/BL6 mice	Partial reduction of oxidative stress and apoptosis through sonic hedgehog signaling pathway.	[192]
92	Chlorogenic acid	Cinnamate ester	*Calluna vulgaris*	120 mg	DSS-induced acute colitis in C57BL/6 mice	Reduces tissue inflammation and apoptosis by a mechanism related to the MAPK/ERK/JNK signaling pathway.	[193]
				40 mg	DSS-induced acute colitis in BALB/c mice	Downregulation of miR-155 expression and inactivation of NF-κB/NLRP3 inflammasome pathway in macrophages.	[194]
93	Tyrosol	Phenylethanoid	olive oil	20 mg	DSS-induced acute colitis in Wistar albino rats	Exerts anti-inflammatory and antioxidant activity.	[195]
94	2,3,5,4′-tetrahydroxystilbene-2-O-beta-D-glucoside	Polyphenol	*Fallopia multiflora*	60 mg	Acetic acid-induced colitis in Kunming mice	Involved in the upregulation of PPAR-γ and inhibition of NF-κB inflammatory pathway.	[196]
95	Resveratrol	Polyphenol	Red grapes	100 mg	TNBS-induced colitis in BALB/c mice	Simultaneous inhibition of inflammatory Th1/Th17 cells through induction of Tregs; regulation of microbiota.	[185]
					DSS-induced chronic colitis in C57BL/6 mice	Reduction of intestinal mucosal barrier dysfunction in UC mice by enhancing autophagy of intestinal epithelial cells.	[184]
					DSS-induced acute colitis in BALB/c mice	Inhibition of PI3K/Akt pathway activation and reduction of VEGFA gene expression.	[183]
96	Salvianolic acid A	Phenolic acids	*Salvia miltiorrhiza*	8 mg	DSS-induced acute colitis in Sprague–Dawley	Reduces intestinal inflammation; regulates the imbalance of intestinal microbiota.	[197]
97	Salvianolic acid B	Phenolic acids	*Salvia miltiorrhiza*	100 mg	DSS-induced acute colitis in C57BL/6 mice	Reduces inflammation; increases the production of short-chain fatty acids; affects the composition of the intestinal microbiota in mice.	[198]

**Table 5:** In vivo therapeutic effects of quinones on experimental IBD.

No.	Name	Type	Origin	Optimal Doses (/kg Body Weight)	Model	Potential Mechanism	References
98	Juglone	Naphthoquinone	*Juglans nigra*	1 mg	DSS-induced ulcerative colitis in ICR mice	Regulation of intestinal microbiota and restoration of Th17/Treg homeostasis.	[18]
99	Emodin	Trihydroxyanthraquinone	*Rheum palmatum*	20 mg	DSS-induced acute colitis in C57BL/6J mice	Increased PPAR-γ expression and inhibited NF-κB activity.	[123]
100	Shikonin	Naphthoquinone	*Echium plantagineum*	25 mg	DSS-induced acute colitis in C57BL/6 mice	Alleviation of inflammation and mucosal barrier damage in UC.	[200]
101	Thymoquinone	Benzoquinone	*Nigella sativa*	40 mg	DSS-induced acute colitis in C57BL/6J mice	Reducing inflammation through the Nrf2/Keap1 system.	[201]
102	Naphthoquinone-2	Naphthoquinone derivative	*Juglans nigra*	100 mg	DSS-induced acute colitis in Wistar rats	Suppression of colonic length, colonic mass index, and intestinal histopathology score.	[202]
103	Plumbagin	Naphthoquinone	*Drosera slackii*	10 mg	DSS-induced acute colitis in C57BL/6J mice	Significantly reduced levels of circulating inflammatory monocytes (CD14+/CD16+) and cytokines (TNF-α and +-IFN-γ).	[203]

**Table 6:** In vivo therapeutic effects of alkaloids on experimental IBD.

No.	Name	Type	Origin	Optimal Doses (/kg Body Weight) or Concentrations	Model	Potential Mechanism	References
104	Capsaicin	Vanilloid	*Capsicum*	12 mg	DSS-induced acute colitis in Sprague–Dawley rats	Inhibits oxidative stress, inflammatory response, and pain signaling.	[211]
105	Matrine	Quinolizidine alkaloid	*Sophora flavescens*	12 mg/mL	TNBS-ethanol-induced ulcerative colitis in Wistar rats	Reduces inflammatory response and oxidative stress damage.	[212]
				20 mg	DSS-induced acute colitis in BALB/c mice	Improves the integrity of the intestinal barrier, inhibits the PPAR-α signaling pathway, and regulates intestinal flora.	[213]
106	Evodiamine	Alkaloid	*Evodia rutaecarpa*	30 mg	DSS-induced chronic colitis in C57BL/6 mice	Reduces the inflammatory response by preventing damage to the intestinal mucosal barrier and regulating the secretion of inflammatory cytokines.	[214]
107	Nuciferine	Alkaloid	*Nymphaea caerulea*	20 mg	DSS-induced acute colitis in BALB/c mice	Regulation of gut microbiota homeostasis and immune function in UC mice.	[215]
108	Piperine	Piperidine alkaloid	*Piper boehmeriifolium*	40 mg	TNBS-induced colitis in Sprague–Dawley rats	Inhibits IκB-α/NF-κB and induces the tight junction proteins claudin-1, occludin, and ZO-1.	[209]
				10 mg	Acetic acid-induced ulcerative colitis in Swiss albino mice	Downregulated the production and expression of inflammatory mediators and reduced the FFA-induced TLR4-mediated inflammatory response.	[208]
109	Nicotine	Alkaloid	Tobacco	10 μg	DSS-induced acute colitis in C57BL/6 mice	Regulates autophagy via AMPK/mTOR pathway; improves inflammation levels.	[40]
110	Neferine	Isoquinoline alkaloid	*Nelumbo nucifera*	10 mg	DSS-induced acute colitis in C57BL/6J mice	Inhibition of inflammatory response.	[216]
				25 mg	DSS-induced acute colitis in C57BL/6 mice	Inhibited iNOS, COX-2, receptor-interacting protein 1 (RIP1), RIP3, and increased caspase-8 protein expression in colonic tissues.	[217]
111	Anatabine	Bipyridines	*Nicotiana cavicola*	20 mg	DSS-induced colitis in C57BL/6 mice	Improves intestinal inflammation and reduces the production of pro-inflammatory factors.	[218]
112	Phellodendrine	Alkaloid	*Phellodendron chinense*	30 mg	DSS-induced acute colitis in C57BL/6 mice	Reduces inflammatory response and promotes autophagy by regulating AMPK-mTOR signaling pathway.	[219]
113	Epiisopiloturine	Imidazole alkaloid	*jaborandi*	1 mg	TNBS-induced colitis in Wistar rats	Downregulation of inflammatory processes by inhibiting the synthesis and release of inflammatory products, lipid peroxidation, and expression of inflammatory enzymes.	[220]
114	Camptothecin	Quinoline alkaloid	*Camptotheca acuminata*	1.5 mg	DSS-induced acute colitis in C57BL/6 mice	Inhibition of inflammatory responses through AKT, NF-κB and MAPK signaling pathways.	[210]
115	Strictosamide	Beta-carboline	*Amsonia orientalis*	40 mg	DSS-induced acute colitis in BALB/c mice	Improving the inflammatory response and NF-κB signaling pathway.	[221]
116	Berberine	Isoquinoline alkaloid	*Berberis vulgaris*	40 mg	DSS-induced acute colitis in BALB/c mice	Regulates the intestinal microbiota and protects the mucosal barrier.	[206]
				100 mg	DSS-induced acute colitis in C57BL/6 mice and Sprague–Dawley rats	Regulation of intestinal glial cell-intestinal epithelial cell-immune cell interactions.	[207]
117	Oxymatrine	Quinolizidine alkaloid	*Sophora pachycarpa*	20 mg	DSS-induced acute colitis in Kunming mice	Reduces inflammatory response and re-establishes antioxidant/oxidant balance.	[222]
				50 mg	DSS-induced acute colitis in BALB/c mice	Inhibition of PI3K/AKT signaling pathway.	[223]
118	Sophocarpine	Quinolizidine alkaloid	*Daphniphyllum oldhamii*	30 mg	DSS-induced acute colitis in BALB/c mice	Maintains the integrity of the colonic barrier and inhibits the development of colitis.	[224]
119	Capnoidine	Tetrahydroisoquinoline alkaloid	*Fumaria capreolata*	/	TNBS-induced acute colitis in C57BL/6 mice	Reduction of colonic histological inflammation.	[225]
120	Oxyberberine	Benzyl tetrahydroisoquinoline alkaloid	*Thalictrum lucidum*	50 mg	DSS-induced acute colitis in BALB/c mice	Influence on the intestinal epithelial barrier, intestinal microbiota, and TLR4-MyD88-NF-κB pathway.	[226]
121	Aloperine	Quinolizidine alkaloid	*Thinicola incana*	40 mg	DSS-induced acute colitis in BALB/c mice	Inhibits the PP2A-Mediated PI3K/Akt/mTOR signaling pathway.	[227]
122	Berberrubine	Benzyl tetrahydroisoquinoline alkaloid	*Coptischinensis*	20 mg	DSS-induced acute colitis in BALB/c mice	Reduction of mucosal lesions and inflammation.	[228]
123	Sinomenine	Morphinane alkaloid	*Sinomenium acutum*	100 mg	DSS-induced acute colitis in C57BL/6 mice	Alleviation of colitis through the Nrf2/NQO 1 signaling pathway.	[229]
124	N-Methylcytisine	Quinolizidine alkaloid	*Thermopsis lanceolata*	16 mg	DSS-induced acute colitis in ICR mice	Inhibition of NF-κB activation.	[230]
125	Tetrandrine	Bisbenzylisoquinoline alkaloid	*Pachygone dasycarpa*	/	DSS-induced acute colitis in mice	Promoting occludin expression through the AhR-miR-429 pathway.	[231]
				40 mg	DSS-induced acute colitis in C57BL/6 mice	Inhibition of NF-κB activation.	[232]
126	Norisoboldine	Benzyl tetrahydroisoquinoline alkaloid	*Cassytha pubescens*	40 mg	TNBS-induced colitis in BALB/c mice	Regulation of the AhR/Nrf2/ROS signaling pathway inhibits NLRP3 inflammasome activation.	[233]
127	Dihydroberberine	Isoquinoline alkaloid	*Thalictrum foliolosum*	50 mg	DSS-induced acute colitis in BALB/c mice	Improved intestinal barrier function; reduced colonic pro-inflammatory cytokines and immunoglobulins by blocking TLR4/MyD88/NF-κB signaling pathway; improved colonic immune inflammation status.	[234]
128	Palmatine	Isoquinoline alkaloid	*Berberis poiretii*	50 mg	DSS-induced acute colitis in BALB/c mice	Inhibition of tryptophan metabolism and regulation of intestinal flora.	[235]
				100 mg	DSS-induced acute colitis in BALB/c mice	Promoting PINK1/Parkin-driven mitochondrial autophagy and thereby inactivating NLRP3 inflammasome in macrophages.	[236]
129	Coptisine	Tetrahydroisoquinoline alkaloid	*Fumaria capreolata*	100 mg	DSS-induced acute colitis in BALB/c mice	Improvement of intestinal barrier dysfunction and inhibition of inflammatory response.	[237]
130	Sanguinarine	Benzophenanthridine alkaloid	*Sanguinaria canadensis*	10 mg	Acetic acid-induced ulcerative colitis in Kunming mice	Effective inhibition of p65 NF-κB protein expression and MPO activity accumulation.	[238]
131	5-Hydroxy-4-methoxycanthin-6-one	Indole alkaloid	*Picrasma quassioides*	100 mg	DSS-induced acute colitis in Sprague–Dawley rats	Regulation of metabolic profile and inhibition of NF-κB/p65 signaling pathway.	[239]`
132	Isatin	Indole alkaloid	*Couroupita guianensis*	25 mg	TNBS-induced colitis in Wistar Hannover rats	Protects the intestinal mucosa from TNBS-induced damage through a combination of antioxidant and anti-inflammatory properties.	[240]
133	Caulerpin	Indole alkaloid	*Caulerpa obscura*	4 mg	DSS-induced acute colitis in C57BL/6 mice	Inhibition of NF-κB pathway activation.	[241]
134	Indirubin	Indole alkaloid	*Isatis tinctoria*	10 mg	DSS-induced acute colitis in BALB/c mice	Inhibition of DSS-induced activation of NF-κB and MAPK pathways.	[242]
				10 mg	DSS-induced acute colitis in BALB/c mice	Inhibition of inflammation and induction of regulatory T cell expression foxp3.	[243]
135	Rutaecarpine	Indole alkaloid	*Tetradium ruticarpum*	80 mg	DSS-induced acute colitis in C57BL/6J mice	Inhibition of KEAP1-NRF2 interaction and activation of NRF2.	[244]
136	Daurisoline	Isoquinoline alkaloid	*Menispermum dauricum*	40 mg	DSS-induced acute colitis in BALB/c mice	Involved in NF-κB and Wnt/β-Catenin pathways.	[245]
137	14-O-acetylneoline	Diterpenoid alkaloid	*Aconitum laciniatum*	/	TNBS-induced colitis in C57BL/6 mice	Reduced IFN-γ mRNA levels in colonic tissues.	[246]

“/” indicates that the reference is not mentioned or is unclear.

**Table 7:** In vivo therapeutic effects of coumarins on experimental IBD.

No.	Name	Type	Origin	Optimal Doses (/kg Body Weight)	Model	Potential Mechanism	References
138	4-methylesculetin	Hydroxycoumarin	*/*	25 mg	DSS-induced acute colitis in Swiss albino mice	Exerts anti-inflammatory properties.	[251]
				5 mg	TNBS-induced colitis in Wistar rats	Reduction of colonic oxidative stress and inhibition of pro-inflammatory cytokine production.	[252]
139	Daphnetin	Hydroxycoumarin	*Euphorbia dracunculoides*	16 mg	DSS-induced acute colitis in BALB/c mice	Regulation of microbiota composition and T reg/T h 17 balance.	[253]
140	Bergapten	Furocoumarin	*Ficus virens*	30 mg	Acetic acid-induced colitis in Sprague–Dawley rats	Reduction of acetic acid-induced inflammation, colonic damage and mast cell degranulation in rats.	[254]
141	Imperatorin	Furocoumarin	*Angelica dahurica*	60 mg	TNBS-induced colitis in Sprague–Dawley rats	Regulation of Nrf-2/ARE/HO-1 pathway in rats.	[255]
142	Osthole	Derivative of coumarin	*Cnidium monnieri*	100 mg	TNBS-induced colitis in C57BL/6 mice	Reducing the expression of inflammatory mediators and decreasing the phosphorylation level of p38.	[248]
				40 mg	DSS-induced acute colitis in BALB/c mice	Blocking the activation of NF-κB and MAPK/p38 pathways.	[249]
143	Esculetin	Hydroxycoumarin	*Artemisia eriopoda*	5 mg	TNBS-induced colitis in Wistar rats	Inhibition of pro-inflammatory cytokine secretion and increased defense against reactive oxygen species.	[256]
144	Umbelliferone	Hydroxycoumarin	*Ficus septica*	30 mg	Acetic acid-induced colitis in Wistar rats	Regulation of TLR4/NF-κB-p65/iNOS and SIRT1/PPARγ signaling pathways in rats.	[250]
145	Imperatorin	Furocoumarin	*Angelica archangelica*	100 mg	DSS-induced acute colitis in C57BL/6 mice	By inhibiting NF-κB-mediated pro-inflammatory responses in a PXR/NF-κB-dependent manner.	[257]
146	Coumarin	Chromenone	*Coumarou*	5 mg	TNBS-induced colitis in Wistar rats	Prevents glutathione depletion due to colonic inflammation.	[258]
147	4-Hydroxycoumarin	Hydroxycoumarin	*Coumarou*	25 mg	TNBS-induced colitis in Wistar rats	Prevents glutathione depletion due to colonic inflammation.	[258]

“/” indicates that the reference is not mentioned or is unclear.

**Table 8:** In vivo therapeutic effects of natural polysaccharides on experimental IBD.

No.	Name	Origin	Optimal Doses (/kg Body Weight)	Model	Potential Mechanism	References
148	Chitosan oligosaccharide	Dietary fiber chitosan	10 mg	DSS-induced acute colitis in ICR mice	Inhibition of NF- B signaling pathway and apoptosis in intestinal epithelial cells.	[270]
149	Mannose	/	500 mg	DSS-induced acute colitis in C57BL/6 mice	Enhanced lysosomal integrity and limited release of histone B; reduced intestinal barrier damage.	[271]
150	*Lycium barbarum* polysaccharides	*Lycium barbarum*	100 mg	DSS-induced acute colitis in Sprague–Dawley rats	Inhibition of oxidative stress, inflammatory response, and pain signaling.	[211]
151	Astragalus polysaccharides	*Astragalus membranaceus*	300 mg	DSS-induced acute colitis in C57BL/6 mice	Inhibits the NRF2/HO-1 pathway.	[263]
			200 mg	DSS-induced acute colitis in C57BL/6 mice	Regulation of Tfh/Treg cell homeostasis.	[264]
152	Chrysanthemum polysaccharides	*Chrysanthemum morifolium*	100 mg	TNBS-induced acute colitis in Sprague–Dawley rats	Promotes the growth of beneficial intestinal flora, regulates the intestinal micro-ecological balance, and restores the immune system.	[272]
			50 mg	TNBS-induced acute colitis in Sprague–Dawley rats	Regulation of metabolic profiles and NF-κ B/TLR4 and IL-6/JAK2/STAT3 signaling pathways.	[273]
153	Garlic polysaccharides	*Allium sativum* L.	400 mg	DSS-induced acute colitis in C57BL/6 mice	Improves the mucosal barrier, blocks pro-inflammatory cytokines, and regulates the intestinal microbiota.	[274]
154	Polysaccharides from *Atractylodes macrocephala* Koidz.	*Atractylodes macrocephala* Koidz.	40 mg	DSS-induced acute colitis in C57BL/6J mice	Modification of intestinal flora and host metabolism to improve UC.	[275]
155	Mannoglucan	Chinese yam	300 mg	DSS-induced acute colitis in C57BL/6J mice	Inhibits excessive production of pro-inflammatory cytokines, suppresses activation of colonic inflammatory signaling pathways, enhances mRNA expression of ligand proteins, and regulates intestinal microbiota.	[276]
156	*Dendrobium fimbriatum* polysaccharides	*Dendrobium fimbriatum*	/	DSS-induced acute colitis in C57BL/6J mice	Improves intestinal barrier function, regulates intestinal flora, and reduces oxidative stress and inflammatory response.	[277]
157	*Ficus carica* polysaccharides	*Ficus carica*	300 mg	DSS-induced chronic colitis in C57BL/6 mice	Improvement of colon length and inhibition of inflammatory cell infiltration in the intestine.	[278]
158	Crude Fuzhuan brick tea polysaccharides	Fuzhuan brick tea	/	DSS-induced colitis in C57BL/6 mice	Regulates intestinal flora, reduces inflammatory response, and improves intestinal barrier function.	[279]
159	*Lonicera japonica Thunb* polysaccharides	*Lonicera japonica Thunb*	150 mg	DSS-induced acute colitis in BALB/c mice	Restoration of immune disorders and improvement of anti-inflammatory activity of immune organs in UC mice.	[280]
160	Noni fruit polysaccharides	*Morinda citrifolia* L.	10 mg	DSS-induced acute colitis in BALB/c mice	Targeting intestinal microbiota regulation; inhibition of JNK, ERK, and NF-κB phosphorylation in IBD mice.	[281]
161	*Scutellaria baicalensis Georgi* polysaccharides	*Scutellaria baicalensis Georgi*	200 mg	DSS-induced acute colitis in C57BL/6 mice	Improvement of intestinal barrier function and regulation of intestinal microbiota.	[282]
162	*Auricularia auricular-judae* polysaccharides	*Auricularia auricular-judae*	40 mg	DSS-induced acute colitis in BALB/c mice	Regulation of the composition of the intestinal microbiota.	[262]
163	Ganoderma lucidum polysaccharides	*Ganoderma lucidum*	/	DSS-induced acute colitis in Wistar rats	Alteration of cecum microbiota and colonic epithelial cell gene expression.	[283]
			100 mg	DSS-induced acute colitis in C57BL/6 mice	Reduces inflammation, maintains intestinal homeostasis, and regulates intestinal immune barrier function.	[284]
164	*Tremella fuciformis* polysaccharides	*Tremella fuciformis*	300 mg	DSS-induced acute colitis in C57BL/6 mice	Regulation of intestinal microbiota and bacterial metabolites.	[267]
165	*Dictyophora indusiata* polysaccharides	*Dictyophora indusiata*	33 mg	DSS-induced acute colitis in BALB/c mice	By improving intestinal damage, oxidative stress and production of pro-inflammatory cytokines; regulating intestinal flora.	[265]
			100 mg	DSS-induced acute colitis in C57BL/6 mice	Reduced oxidative stress and inflammatory response, inhibited key signaling pathways associated with colitis, improved expression of tight junction proteins, and downregulated polarization of M1 macrophages.	[285]

“/” indicates that the reference is not mentioned or is unclear.

**Table 9:** Therapeutic effects of natural proteins and active peptides on experimental IBD.

No.	Name	Origin	Amino Acid Sequence	Optimal Doses (/kg Body Weight)	Model	Potential Mechanism	References
166	Soy tripeptide	soy	VPY	100 mg	DSS-induced acute colitis in BALB/c mice	Downregulation of pro-inflammatory cytokine expression in the colon and amelioration of inflammation in colonic tissue.	[287]
167	Pyroglutamyl leucine	wheat gluten hydrolysate	pyroGlu-Leu	0.1 mg	DSS-induced acute colitis in C57BL/6 mice	Regulation of intestinal microorganisms.	[288]
168	Phycocyanin	*Aphanizomenon flos-aquae*	/	/	DSS-induced acute colitis in C57BL/6 mice	Protects the intestinal epithelial barrier; anti-inflammatory and antioxidant.	[289]
169	Melittin peptide	*Apis mellifera*	GIGAVLKVLTTGLPALISWIKRKRQQ	2.4 mg	DSS-induced acute colitis in C57BL/6 mice	Eliminates histological damage to colonic tissue and reduces inflammation. Regulates oxidative/antioxidant balance.	[290]
			/	40 μg	Acetic acid-induced colitis in Swiss albino mice	Attenuates TLR4/TRAF6-mediated activation of NF-κB and p38MAPK pathways.	[295]
170	Yellowtail milt hydrolysates	*Seriola quinqueradiata*	/	/	DSS-induced acute colitis in C57BL/6 mice	Improvement of colitis symptoms and intestinal epithelial barrier dysfunction in mice.	[291]

“/” indicates that the reference is not mentioned or is unclear.

## Data Availability

Not applicable.
